# Supplementary material for: Combined analyses of within-host SARS-CoV-2 viral kinetics and information on past exposures to the virus in a human cohort identifies intrinsic differences of Omicron and Delta variants
Source: PLoS Biol. 2024 Jan 30;22(1):e3002463. doi: 10.1371/journal.pbio.3002463 (PMC10826969; doi:10.1371/journal.pbio.3002463)
Supplement: S1 Table — We present a table of summary statistics, including the number of points available per infection episode, the delay between first viral load data and symptom onset, the observed peak viral load, and the time until clearance (approximated by the final time point per infection episode) for all individuals that had not had an infection before joining the study (N = 75). (DOCX) [file pbio.3002463.s001.docx]

| **Infection naïve individuals (N = 75)** | **VOC** | | |
| --- | --- | --- | --- |
|  | **Delta** | **Omicron (BA.1)** | **Omicron (BA.2)** |
| **Number of PCR test results** | 4.0 [1.4 — 6.6] | 4.0 [0.38 — 7.6] | 5.0 [1.0 — 9.0] |
| **Time of symptom onset (days since first positive PCR test)** | 1.0 [-0.38—2.4] | 0.0 [-1.9—1.9] | -1.0 [-2.5—0.5] |
| **Observed peak Ct value** | 18.7 [15.8—21.5] | 18.8 [16.5—21.2] | 17.0 [15.3—18.8] |
| **Time of final positive PCR test result** | 9.0 [6.9—11] | 9.5 [6.5—12] | 12.0 [8.5—16] |
|  | | | |
| **Previously infected individuals on entry to the study (N = 42)** | **VOC** | | |
|  | **Delta** | **Omicron (BA.1)** | **Omicron (BA.2)** |
| **Number of PCR test results** | 3.0 [0.38 — 5.6] | 4.0 [0.5 — 7.5] | 6.0 [2.0 — 10.0] |
| **Time of symptom onset (days since first positive PCR test)** | 0.0 [-3.2—3.2] | -1.0 [-3.1—1.1] | -0.5 [-1.6—0.62] |
| **Observed peak Ct value** | 18.9 [15.0—22.7] | 17.7 [15.4—20.1] | 17.1 [15.0—19.2] |
| **Time of final positive PCR test result** | 7.5 [4.8—10] | 7.0 [5.8—8.2] | 9.0 [4.0—14] |
|  | | | |
| **Previously infected individuals on entry to the study (N = 152)** | **VOC** | | |
|  | **Delta** | **Omicron (BA.1)** | **Omicron (BA.2)** |
| **Number of PCR test results** | 4.0 [1.4 — 6.6] | 4.0 [0.38 — 7.6] | 5.0 [1.0 — 9.0] |
| **Time of symptom onset (days since first positive PCR test)** | 1.0 [-0.38—2.4] | 0.0 [-1.9—1.9] | -1.0 [-2.5—0.5] |
| **Observed peak Ct value** | 18.7 [15.8—21.5] | 18.8 [16.5—21.2] | 17.0 [15.3—18.8] |
| **Time of final positive PCR test result** | 9.0 [6.9—11] | 9.5 [6.5—12] | 12.0 [8.5—16] |
