# Supplementary material for: Combined analyses of within-host SARS-CoV-2 viral kinetics and information on past exposures to the virus in a human cohort identifies intrinsic differences of Omicron and Delta variants
Source: PLoS Biol. 2024 Jan 30;22(1):e3002463. doi: 10.1371/journal.pbio.3002463 (PMC10826969; doi:10.1371/journal.pbio.3002463)
Supplement: S2 Table — We list each parameter within our modelling framework, its biological interpretation, the prior distribution we chose for it, and any references justifying the choice of prior. (DOCX) [file pbio.3002463.s002.docx]

| **Parameter** | **Description** | **Prior/equation** | **Notes** |
| --- | --- | --- | --- |
| $\bar{t_{p}}$ | Covariate-level time of peak | $\text{Normal}(\text{log}(5), 0.5)$ | Mean at 5 days after exposure, on log scale |
| $\bar{t_{\text{lod}}}$ | Covariate-level time the latent limit of detection is reached | $\text{Normal}(\text{log}(25), 0.5)$ | Mean at 25 days after exposure, on log scale |
| $\bar{c_{e}}$ | Covariate-level Ct value at exposure | $\text{Normal}(50, 5) \text{T}[40, 0]$ | Mean at 50 truncated at experimental LOD of 40 |
| $\bar{c_{p}}$ | Covariate-level Ct value of peak | $\text{Normal}(0, 1)$ | Peak at 50% of exposure value, on logit scale |
| $\bar{c_{\text{lod}}}$ | Covariate-level Ct value of latent limit of detection | $\bar{c_{\text{lod}}}$ = $\bar{c_{e}}$ | Latent exposure and limit of detect Ct value assumed to be the same |
| $t_{e}$ | Individual-level timing of exposure | $\text{Normal}\left( t_{\bar{\text{inf}}}+5, 5 \right)$  $t_{\bar{\text{inf}}}=max({-t}_{\text{onset}}, 0)$ | Timing of exposure at mean of lower bound for infection time, given by either time of onset or first positive test |
| $t_{p}$ | Individual-level timing of peak | $t_{p} = \text{exp}(\beta^{t_{p}}\bar{t_{p}} + \eta_{t_{p}})$ | Non-centred parameterisation of peak timing, with regression coefficient and individual-level variation, on exponential scale |
| $t_{lod}$ | Individual-level timing limit of detection reached | $t_{\text{lod}} = \text{exp}(\beta^{t_{\text{lod}}}\bar{t_{lod}} + \eta_{t_{\text{lod}}})$ | Non-centred parameterisation of LOD, with regression coefficient and individual-level variation, on exponential scale |
| $c_{e}$ | Individual-level Ct value at exposure | $c_{e} = \beta^{c_{e}}\bar{c_{e}} + \eta_{c_{e}}$ | Non-centred parameterisation of latent Ct value at exposure timing, with regression coefficient and individual-level variation |
| $c_{p}$ | Individual-level Ct value at peak | $c_{p} = \text{logit}^{-1}(\beta^{c_{p}}\bar{c_{p}} + \eta_{c_{p}})$ | Non-centred parameterisation of latent peak Ct value, with regression coefficient and individual-level variation, on logit scale |
| $c_{lod}$ | Individual-level Ct value at limit of detection | $c_{\text{lod}} = c_{e}$ | Non-centred parameterisation of latent Ct value at LOD, with regression coefficient and individual-level variation |
| $\boldsymbol{\beta}$ | Covariate-level effect size parameters | $\beta_{i}\boldsymbol{\sim}\text{Normal}\boldsymbol{(}0\boldsymbol{,} 0.025\boldsymbol{)}$ | Vector of regression coefficients, specified by formula before fitting model, relative to baseline set of individuals |
| $\eta$ | Individual-level variation | $\eta_{p} \sim\text{MVN}(0, \boldsymbol{\Sigma})$ $\boldsymbol{\Sigma}=\boldsymbol{\Delta\Omega\Delta}$ $\boldsymbol{\Omega}\boldsymbol{\sim}\text{LKJCorr}(1)$ $\sigma_{p} \sim\text{Half-Normal}(0, 0.2)$ | Cholesky decomposition of multivariate normal distribution to control individual-level variation, with LKJ prior over correlation matrix |
