# Supplementary material for: Combined analyses of within-host SARS-CoV-2 viral kinetics and information on past exposures to the virus in a human cohort identifies intrinsic differences of Omicron and Delta variants
Source: PLoS Biol. 2024 Jan 30;22(1):e3002463. doi: 10.1371/journal.pbio.3002463 (PMC10826969; doi:10.1371/journal.pbio.3002463)
Supplement: S3 Table — (DOCX) [file pbio.3002463.s003.docx]

|  | **VOC** | | |
| --- | --- | --- | --- |
|  | **Delta** | **BA.1 (baseline)** | **BA.2** |
| **Peak Ct value** | 14.8 (13.5—16.2) | 15.9 (14.8—16.9) | 14.9 (13.9—16.0) |
| **Timing of the peak (days)** | 6.6 (5.1—8.3) | 5.9 (5.2—6.7) | 5.2 (4.3—6.1) |
| **Time until PCR -ve (days)** | 21.7 (19.2—24.7) | 24.2 (21.9—26.6) | 25.3 (22.7—28.0) |
