# Supplementary material for: Combined analyses of within-host SARS-CoV-2 viral kinetics and information on past exposures to the virus in a human cohort identifies intrinsic differences of Omicron and Delta variants
Source: PLoS Biol. 2024 Jan 30;22(1):e3002463. doi: 10.1371/journal.pbio.3002463 (PMC10826969; doi:10.1371/journal.pbio.3002463)
Supplement: S4 Table — (DOCX) [file pbio.3002463.s004.docx]

|  | **Symptom status** | |
| --- | --- | --- |
|  | **Symptomatic (baseline)** | **Asymptomatic** |
| **Peak Ct value** | 15.9 (14.8—16.9) | 16.6 (15.1—18.2) |
| **Timing of the peak (days)** | 5.9 (5.2—6.7) | 5.5 (4.2—6.9) |
| **Time until PCR -ve (days)** | 24.2 (21.9—26.6) | 21.8 (19.1—25.0) |
