# Supplementary material for: Combined analyses of within-host SARS-CoV-2 viral kinetics and information on past exposures to the virus in a human cohort identifies intrinsic differences of Omicron and Delta variants
Source: PLoS Biol. 2024 Jan 30;22(1):e3002463. doi: 10.1371/journal.pbio.3002463 (PMC10826969; doi:10.1371/journal.pbio.3002463)
Supplement: S5 Table — (DOCX) [file pbio.3002463.s005.docx]

|  | **Total number of exposures** | | |
| --- | --- | --- | --- |
|  | **3** | **4 (baseline)** | **5+** |
| **Peak Ct value** | 17.0 (15.3—18.8) | 15.9 (14.8—16.9) | 18.2 (16.9—19.6) |
| **Timing of the peak (days)** | 5.3 (4.2—6.6) | 5.9 (5.2—6.7) | 5.9 (4.7—7.2) |
| **Time until PCR -ve (days)** | 21.6 (18.7—25.1) | 24.2 (21.9—26.6) | 19.3 (17.2—21.6) |
