# Supplementary material for: Combined analyses of within-host SARS-CoV-2 viral kinetics and information on past exposures to the virus in a human cohort identifies intrinsic differences of Omicron and Delta variants
Source: PLoS Biol. 2024 Jan 30;22(1):e3002463. doi: 10.1371/journal.pbio.3002463 (PMC10826969; doi:10.1371/journal.pbio.3002463)
Supplement: S6 Table — (DOCX) [file pbio.3002463.s006.docx]

|  | **Age** | | |
| --- | --- | --- | --- |
|  | **20—34** | **35—49 (baseline)** | **50+** |
| **Peak Ct value** | 16.0 (14.9—17.2) | 15.9 (14.8—16.9) | 17.7 (16.4—18.9) |
| **Timing of the peak (days)** | 4.5 (3.7—5.4) | 5.9 (5.2—6.7) | 6.2 (5.2—7.2) |
| **Time until PCR -ve (days)** | 24.9 (22.2—28.3) | 24.2 (21.9—26.6) | 27.5 (24.4—30.0) |
