# Supplementary material for: Combined analyses of within-host SARS-CoV-2 viral kinetics and information on past exposures to the virus in a human cohort identifies intrinsic differences of Omicron and Delta variants
Source: PLoS Biol. 2024 Jan 30;22(1):e3002463. doi: 10.1371/journal.pbio.3002463 (PMC10826969; doi:10.1371/journal.pbio.3002463)
Supplement: S7 Table — The models are reported in no particular order. (DOCX) [file pbio.3002463.s007.docx]

| **Model description** | **Regression formula** | **ELDP-LOO** | **ELDP-diff** | **SE** | **SE-diff** |
| --- | --- | --- | --- | --- | --- |
| Main model | ~ 1 + VOC + symptom status + number of exposures + age group + time since last exposure | -3386.9 | -43.7 | 49.4 | 14.0 |
| VOC | ~ 1 + VOC | -3359.4 | -16.2 | 51.1 | 9.5 |
| Symptom status | ~ 1 + symptom status | -3349.3 | -6.1 | 53.2 | 13.4 |
| Exposures | ~ 1 + number of exposures | -3419.6 | -76.4 | 60.9 | 28.2 |
| Age group | ~ 1 + age group | -3343.2 | 0.0 | 48.3 | 0.0 |
| No VOC | ~ 1 + symptom status + number of exposures + age group + time since last exposure | -3366.8 | -23.6 | 46.7 | 8.3 |
| No covariates | ~ 1 | -3368.3 | -25.1 | 55.9 | 18.3 |
| Uninformative priors | ~ 1 + VOC + symptom status + number of exposures + age group + time since last exposure | -3376.0 | -32.8 | 48.2 | 8.0 |
