# Supplementary material for: Combined analyses of within-host SARS-CoV-2 viral kinetics and information on past exposures to the virus in a human cohort identifies intrinsic differences of Omicron and Delta variants
Source: PLoS Biol. 2024 Jan 30;22(1):e3002463. doi: 10.1371/journal.pbio.3002463 (PMC10826969; doi:10.1371/journal.pbio.3002463)
Supplement: S1 Text — Full supporting information, including all supporting figures and detailed methods. Supporting tables are included as separate files. (PDF) [file pbio.3002463.s008.pdf]

Supporting information for:

Combined analyses of within-host SARS-CoV-2 viral kinetics and information on past exposures to the virus in a human cohort identifies intrinsic differences of Omicron and Delta variants

Table of Contents

Supporting information for: *1*

Combined analyses of within-host SARS-CoV-2 viral kinetics and information on past exposures to the virus in a human cohort identifies intrinsic differences of Omicron and Delta variants *1*

Detailed description of the data **4**
Cycle threshold data: ORF1ab, N and S-gene targets 4

Detailed methods **10**
Model overview and description 10

Viral kinetic Ct model **11**

Likelihood function terms **11**
Viral kinetics likelihood function 11
Incubation period likelihood term 12

Statistical model structure **14**
Viral kinetics model 14
Individual-level variation 16
Bayesian linear regression structure 17
Gene target and swab type adjustment 17

Individual-level posterior and posterior predictive distributions **18**

Computational details **18**

Timing parameters **19**
Delta-infected individuals 19
Omicron (BA.1)-infected individuals 20
Omicron (BA.2)-infected individuals 21

Viral kinetics model fits **23**
Delta-infected individuals 23
Omicron (BA.1)-infected individuals 24
Omicron (BA.2)-infected individuals 25

Timing parameters, prior vs posterior **26**
Delta-infected individuals 26
Omicron (BA.1)-infected individuals 27
Omicron (BA.2)-infected individuals 28

Timing of rapid test positivity **29**

Tables of population-level posterior estimates **30**

Sensitivity analysis with alternative model fits **31**
No covariates other than VOC 32
Greater individual-level variation 33
No correlation between individual-level parameters 34
No symptom onset data used in likelihood 35
Less informative priors 36

Alternative Ct threshold for Figure 4 ..... **38**

Incubation period estimates **39**

Gene target and swab type adjustment **40**

Population-level prior vs posterior comparison **41**

Simulation study 42
Prior predictive distribution 45
Leave-one-out model comparison 46
References 47

Detailed description of the data

Cycle threshold data: ORF1ab, N and S-gene targets

For most swab tests performed during the study, multiple SARS-CoV-2 genes were targeted. ORF1ab, N-gene and S-genes were tested for. Figures S1 and S2 show the frequency of testing for all individuals along with which genes were targeted for which individuals for each test and the resulting Ct value (respectively). In order to reliably fit to as much data as available (i.e., when available, Ct values for all three targets), we investigated the relationship between the Ct values taken at the same time point as each other for the three different targets.

Close to linear relationships across the range of Ct values for combinations of two of the three targets were observed (**Fig C**). As such, we included a Ct target adjustment component to the statistical component of the model, whereby a linear relationship was assumed between each of the three targets and parameters of a linear regression were estimated within the same framework as the rest of the model. As such, the posteriors for the Ct target adjustment model were propagated through the rest of the inference framework. Details of the in-model adjustment used to adjust for gene target and swab type are included in the detailed methods section.

Over half of the individuals within the study had not reported an infection before they entered the study. This of course does not necessarily mean they had not had an infection, especially as a high proportion of infections during the relevant waves were asymptomatic. However, assuming they were indeed infection naïve, they could have different viral load dynamics to previously infected individuals. We do account for this, given that we use both: the total number of exposures (the sum of infections and vaccines for each individual), and the time since the most recent exposure for each individual as covariates in our model. However, given that the exposures were treated equally, we did not focus solely on infection naïve individuals. To investigate any such differences and to highlight this subpopulation, we include a table and a figure of the summary statistics for the infection naïve individuals (**S1 Table**) plotting the following: the number of points available per patient, the delay between first viral load data and symptom onset, the observed peak viral load, and the time to clearance, all stratified by the VOCs considered in this study (**Fig D)**.

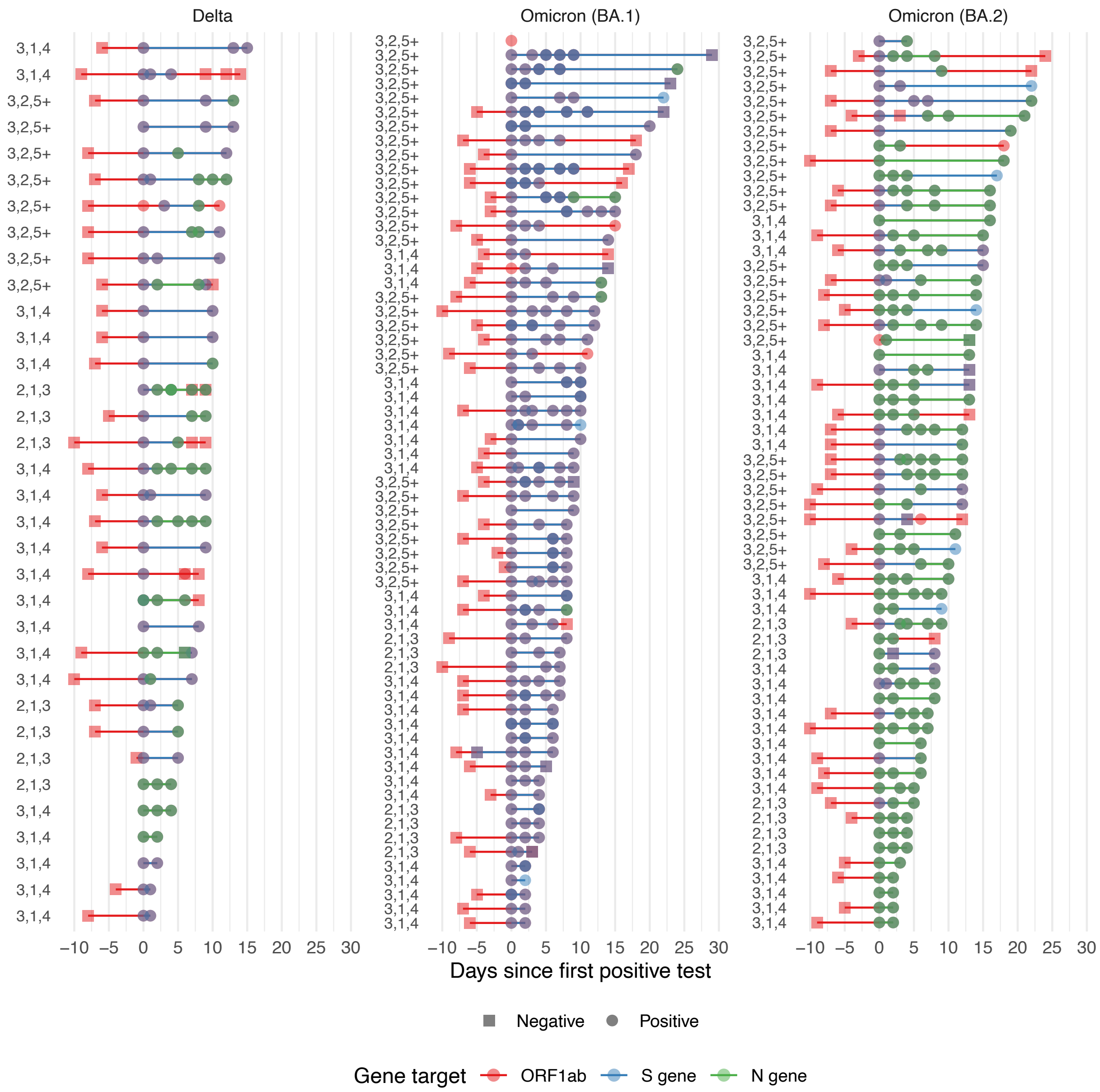

Fig A. Frequency of testing and which gene targets were detected for each infection episode included in the inference. We plot the PCR test results over time to illustrate the testing frequency and which of the three potential gene targets were detected, for each infection episode with two or more positive tests. Mixed colours indicate more than one gene target was detected for a particular PCR test. The figure is of the same format as **Fig B**, panel B. The only change being the colour indicates which gene targets were detected, rather than the Ct value recorded.

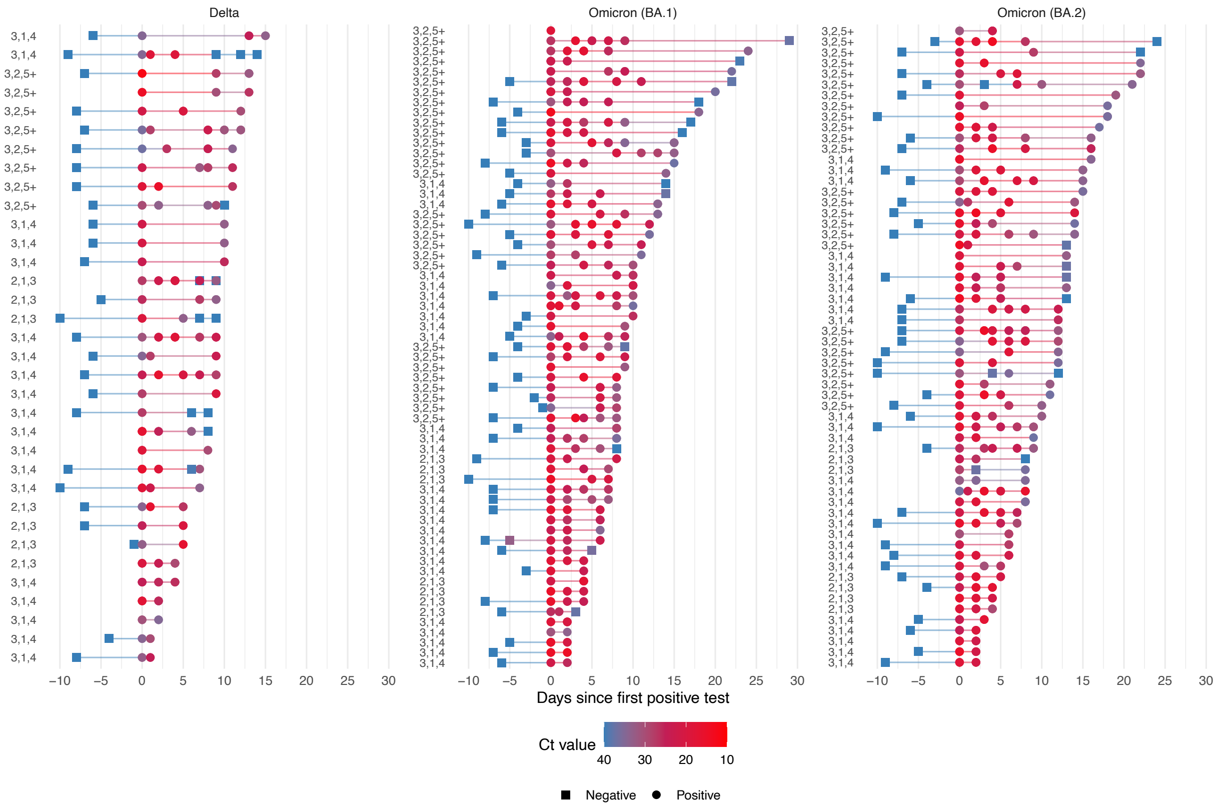

Fig B. Frequency of testing and the resulting Ct value of each test. We plot the PCR test results over time to illustrate the testing frequency and the resulting Ct value associated with each test. The figure is of the same format as **Fig A**. The only change being the colour indicates the Ct value, rather than which gene targets were detected.

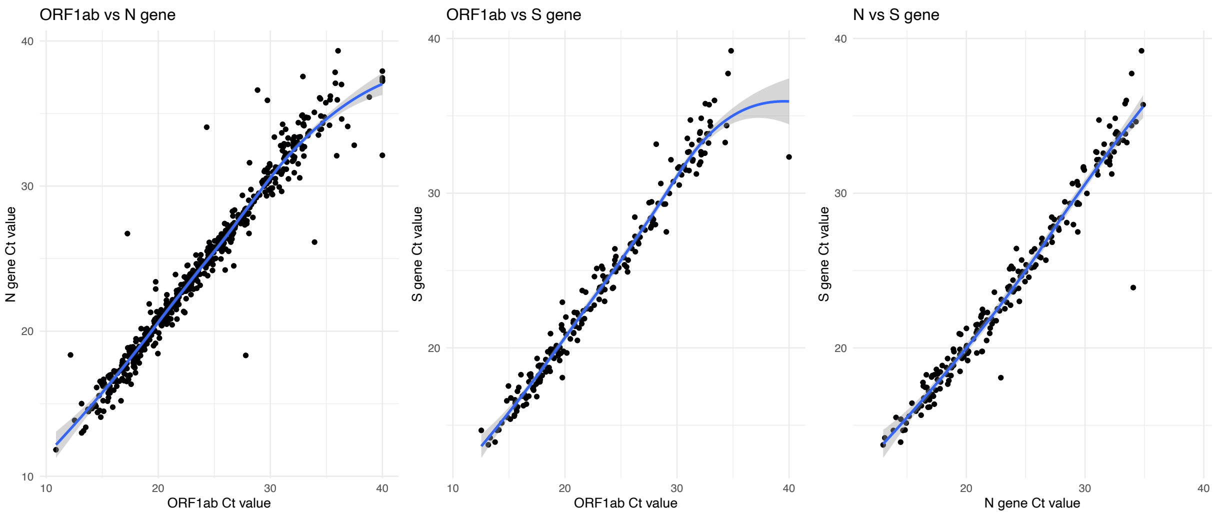

Fig C. Scatterplots between all combinations of the three gene targets for which Ct values were available, with fitted LOESS curves and uncertainty. We plot the Ct values at each pair of target sites against each other to investigate their relationships and the certainty of the relationships. We do so to inform the model specification required to adjust for the gene target used.

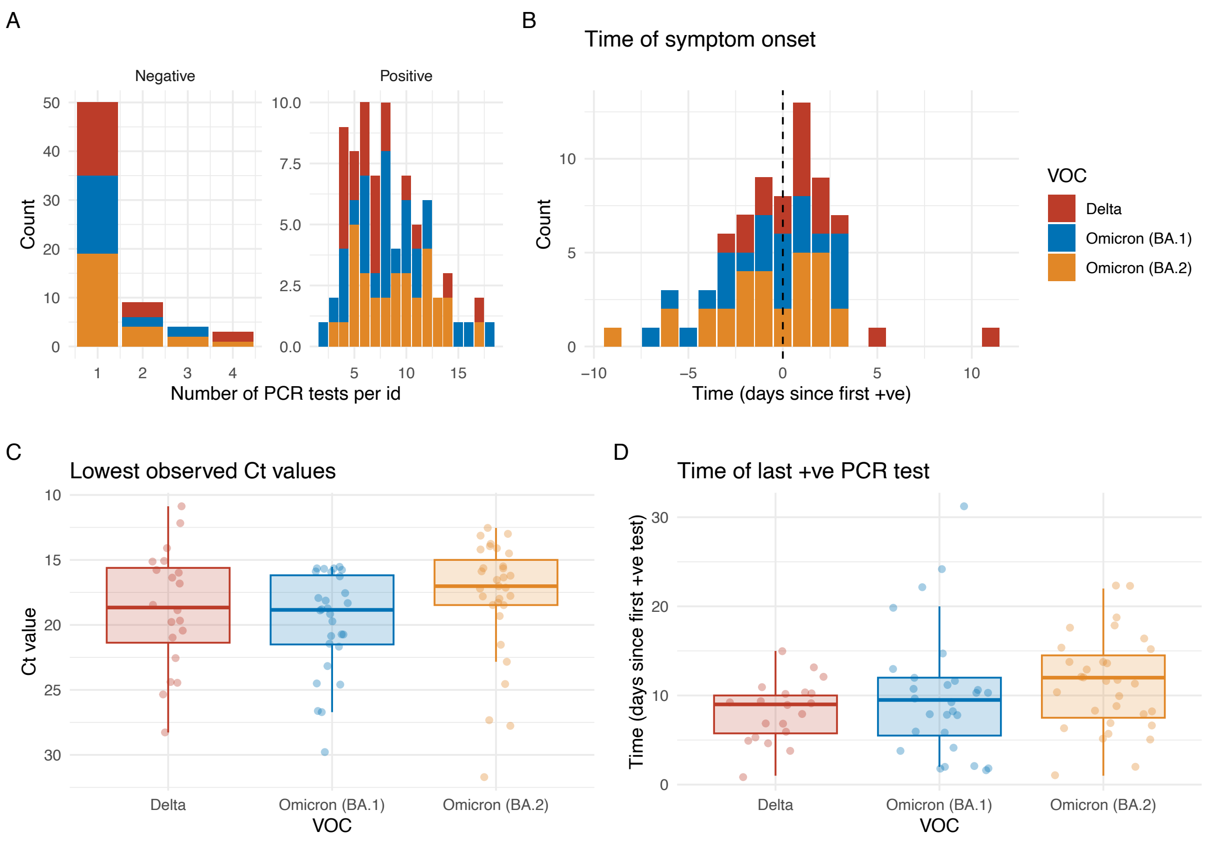

Fig D. Summary statistics for the subpopulation which were infection naïve at the start of the study. A Histogram of the number of PCR tests, stratified by positivity and VOC. **B** Histogram of the time difference between symptom onset and the first positive test in days, stratified by VOC. **C** Boxplot of the lowest observed (highest viral load) Ct values, representing a crude summary of the peak Ct values, stratified by VOC. **D** A boxplot of the time in days between the first and last positive tests for each individual, stratified by VOC. A crude statistic for the time individuals remained detectable by PCR.

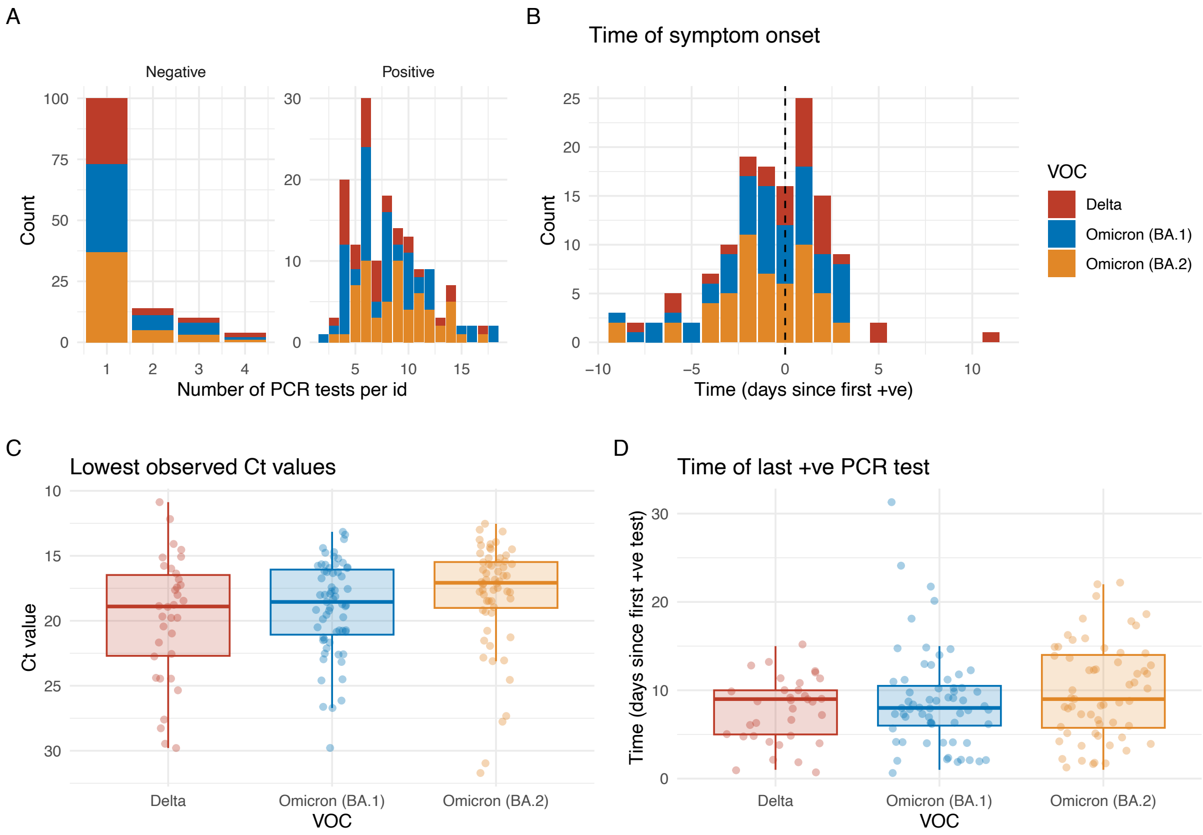

Fig E. Summary statistics for the entire cohort. We plot the same summary statistics in the same format as **Fig D,** for the entire cohort for comparison.

Detailed methods

Model overview and description

The biological components of the model are the *within-host viral kinetics model*, fit to longitudinal Ct data for each individual; and the *incubation period model*, fit to individual-level symptom onset data. The statistical components are a Bayesian linear regression structure used to estimate the multiplicative effect between each group of individuals and the chosen baseline set of covariates; and the hierarchical structure of the model, allowing estimation of the covariate-level effects by pooling the individual-level estimates together along with a multivariate normal distribution allowing correlations to be estimated and controlled between the individual-level parameters of the same type. Here, we give a brief technical description of each component:

- The viral kinetic Ct kinetics model calculates the expected Ct at any time, given a set of individual-level parameters, including an unobserved exposure time parameter used in other terms of the likelihood function.
- The measurement model relates each observed Ct value to an expected Ct value with measurement error which we assume is normally distributed. This term is conditioned on the individual-level longitudinal Ct values.
- The component which estimates the incubation period assumes a log-normally distributed incubation period, in line with most of the previously estimates incubation periods for SARS-CoV-2. We fit shape and scale parameters for each set of covariates. This component of the model also depends on the latent exposure time parameter from the viral kinetic model component. This term is conditioned on the individual-level symptom onset time (relative to first positive test).
- The multiple linear regression model, which is used to regress the viral kinetic model and incubation period parameters against an arbitrary selection of covariates. Specifically, effect size parameters are estimated describing the multiplicative change in the five trajectory parameters and the two incubation period parameters between covariates.
- The gene target and swab type adjustment model. We assume a linear relationship, based on plotting the raw data (**Fig C**) for the adjustment model between different gene targets.
- Lastly, the hierarchical structure of the model, which see the individual-level parameters drawn from population-level distributions. The population-level distributions are given semi-informative priors from previous studies (see **Figs 1B** and **1C** for a schematic of the hierarchical structure of the model). Correlations between individual-level parameters are controlled using a multivariate normal distribution.

Viral kinetic Ct model

We used a reparameterised and reformulated version of a previously developed semi-parametric model, used in several other studies of SARS-CoV-2 viral kinetics (1–3). The model assumes a linear increase, a spline point representing the peak of viral shedding and a linear decrease on the cycle threshold scale. Given the relationship between Ct values and viral load, this represents an exponential increase and decrease (with two separate rates) on the viral load scale.

The Ct kinetics model is given by

$$g(t;\,\boldsymbol{\theta}) = \begin{cases} c_e, & t \le t_e \\ \dfrac{(t-t_e)(c_p-c_e)}{t_p} + c_e, & t_e < t \le t_e + t_p \\ \dfrac{(t-t_e-t_p)(c_{\text{lod}}-c_p)}{t_{\text{lod}}} + c_p, & t_e + t_p < t \le t_e + t_p + t_{\text{lod}} \\ c_e, & t > t_e + t_p + t_{\text{lod}}. \end{cases}$$

where t_e is time the individual was exposed, t_p is the time at which the individual's Ct trajectory peaks, t_{lod} is the time at which the trajectory hits the limit of detection, c_e is the theoretical Ct value at exposure, c_p is the Ct value at the peak of the trajectory, and c_{lod} is the theoretical value at which a near-perfect PCR machine would no longer detect any virus. Lastly $\boldsymbol{\theta}$ is a vector of the process parameters for the viral kinetics model. Specifically, $\boldsymbol{\theta} = (t_e,\ t_p,\ t_{\text{lod}},\ c_e,\ c_p,\ c_{\text{lod}})$.

Likelihood function terms

Viral kinetics likelihood function

The log-likelihood for this component is given by

$$L_{\text{Ct}}(\boldsymbol{\theta};\,\boldsymbol{y_t}) = \log\Big(f(y_t \mid g(t), \sigma^2) - \Phi^{-1}(0 \mid g(t), \sigma^2) + \Phi^{-1}\big(c_{\text{lod}} \mid \overline{g(t)}, \sigma^2\big)\Big)$$

where $\boldsymbol{y_t}$ represents the observed each Ct value is assumed to be independent and identically distributed and the variance, σ^2, is estimated in our inference framework, representing the measurement error involved in the PCR process. f(x | y) represents the probability density function for a Normal distribution. As such, we assume that the observed Ct values are normally distributed, with a shared noise term σ^2 across all covariates and individual,

representing the noise inherent in measuring Ct values from a PCR machine.

The other two terms in the likelihood function correspond to adjusting for the fact that the observed Ct values were censored above at Ct = 40 and truncated below at Ct = 0. Specifically, the second and third terms correspond to complementary cumulative distribution functions which deal with these censored values in a relatively standard way within Bayesian inference frameworks. Specifically, $\Phi^{-1}(\mathrm{y} \mid g(t), \sigma^2)$ represents the complementary cumulative density function of the normal distribution for a given Ct value y, with the underlying viral kinetics model at time t as its mean, and the same error term as the non-censored term of the likelihood function σ^2 as its variance. The third term corresponds to the adjustment for Ct values generated by the underlying viral kinetics model which exceed the upper censoring bound, which we denote with $\overline{g(t)}$. The limit of detection parameter, c_{lod}, is a latent parameter to be estimated. This approach deals with the lower truncation and upper censoring in a statistically robust way and is an extension to previous studies which used similar underlying models but without fully accounting for censoring and truncation or modelling the underlying latent time of infection and latent limit of detection.

Incubation period likelihood term

To estimate the incubation period, we first calculate the difference between the date of the estimated infection/exposure time and the symptom onset date for each individual. We then form a censored interval of onset times of 24 hours, given that a single date could represent any time within a 24-hour period. We denote the lower and upper limits of the censored time interval with O_{lower} and O_{upper}, respectively.

We assume a log-normally distributed incubation period, a common parametric choice when estimating the incubation period of SARS-CoV-2 (4). We then use the following term contributing to the overall log-likelihood function for estimating the parameters, μ_{IP} and σ_{IP}, of the log-normal distribution:

$$L_{IP} = \log(F_{IP}(O_{\text{upper}} \mid I_{\mu}, I_{\sigma}^2) - F_{IP}(O_{\text{lower}} \mid I_{\mu}, I_{\sigma}^2)),$$

where the F_{IP} terms denote the cumulative distribution function of the log-normal distribution, I_{μ} and I_{σ}^2 correspond to the location and scale parameters of the log-normal distribution, which we estimate in our framework, and the O_{upper} and O_{lower} terms refer to the lower and upper bounds on the censored interval of the time of symptom onset (in days). We describe all of the parameters and choices for their prior distributions in **S2 Table**. However, for some

context, we briefly describe the choice of priors for the incubation period term here.

The following distributions were chosen as priors for the covariate-level incubation period parameters

$$I_{\mu} \sim \text{Normal}(\mu_{I\mu}, \sigma_{I\mu})$$
$$I_{\sigma} \sim \text{Normal}(\mu_{I\sigma}, \sigma_{I\sigma})$$

here I_{μ} and I_{σ} are the mean and standard deviation of the assumed log-normal distribution of the latent incubation period. As these are parameters of a log-normal distribution, the values given should be interpreted on the exponential scale. Doing so gives the probability that symptom onset relative to the estimated time since exposure. For example, the mean of the prior for I_{mu} corresponds to an incubation period of approximately 5 days since exposure, when exponentiated and interpreted on the natural scale (i.e., $\exp(1.621) \approx 5.06$ days).

Statistical model structure

Viral kinetics model

The model has the following statistical structure, whereby $\bar{\theta}$ represents a population-level parameter and θ represents the corresponding individual-level parameter:

$$\overline{t_e} \sim \text{Normal}(\mu_{t_e}, \sigma_{t_e})$$
$$\overline{t_p} \sim \text{Normal}(\mu_{t_p}, \sigma_{t_p})$$
$$\overline{t_{\text{lod}}} \sim \text{Normal}(\mu_{t_{\text{lod}}}, \sigma_{t_{\text{lod}}})$$
$$\overline{c_e} \sim \text{Normal}(\mu_{c_e}, \sigma_{c_e})$$
$$\overline{c_p} \sim \text{Normal}(\mu_{c_p}, \sigma_{c_p})$$
$$\overline{c_{\text{lod}}} = c_e$$
$$t_e = \exp(\beta_{t_e}\overline{t_e} + \eta_{t_e})$$
$$t_p = \exp\left(\beta_{t_p}\overline{t_p} + \eta_{t_p}\right)$$
$$t_{\text{lod}} = \exp(\beta_{t_{\text{lod}}}\overline{t_{\text{lod}}} + \eta_{t_{\text{lod}}})$$
$$c_e = \beta_{c_e}\overline{c_e} + \eta_{c_e}$$
$$c_p = \text{logit}^{-1}(\beta_{c_p}\overline{c_p} + \eta_{c_p})$$
$$c_{\text{lod}} = c_e$$
$$\beta_i \sim \text{Normal}(0, \sigma_\beta)$$
$$\eta_p \sim \text{MVN}(0, \mathbf{\Sigma})$$

The six process parameters of the viral kinetics model: $t_e, t_p, t_{\text{lod}}, c_e, c_p$ and c_{lod} are modelled with random effects. That is to say that we model the viral kinetics on the individual scale, drawing each individuals' parameters from population-level priors. The individual-level parameters include effect size parameters denoted by β. These measure the relative effect of each of the covariates (VOC, symptom status, age and number of exposures) on each of the process parameters. The population-level priors are denoted by barred versions of the individual-level parameters. The individual-level variation, controlled by the η parameter is described in more detail in the next section. The Ct value at time t for individual i is given by ${y^i}_t$, the likelihood function for which is given in more detail in the previous section *Likelihood function terms*. The adjustments for censoring and truncation of the Ct value data is omitted from this description for brevity, as it is described fully in the previous section.

The process parameters c_{e} and c_{lod} represent the Ct value at the moment of exposure. This is a latent quantity as the virus would not be practically detectable by any realistic PCR

machine at this time. However, it helps us frame and interpret the model by including it for each individual, as it allows us to explicitly model the timing of exposure for each individual. These parameters differ from the LOD of the PCR machine used, which was Ct = 40.

The overall model structure can be described as a two-level, hierarchical model, with a multivariate normal distribution (η) to allow for individual-level parameters to be correlated and effect size parameters (β). We describe how we implement the multivariate normal distribution in more detail in the following section. We chose a non-centred parameterisation for the individual-level parameters, following the guidance from the Stan user guide, to help with sampling from a high-dimensional posterior distribution with complicated geometry.

For brevity, the censoring adjustments within the term relating the observed Ct values to the viral kinetic model are omitted. Furthermore, the term relating the symptom onset dates to the incubation period parameters are omitted, as they are already given in the previous section about the incubation period model structure. Lastly, the details about the specific implementation of the multivariate normal structure are left until the next section.

The individual-level value t_{bound} represents the censored interval in which infection must have occurred, based on each individual's symptom onset time (relative to their first positive test). Specifically, $t_{\text{bound}} = \max(-t_{\text{onset}}, 0)$.

These semi-informative priors were chosen by combining estimates from the human challenge study (Killingley et al. (2022) (5)) and estimates of the viral kinetics from previous studies (Hay et al. (2022) (1)). We have little prior knowledge of each individual's exposure time, which is why the prior for this parameter at the population level was wide in comparison to the other priors. The remaining parameters correspond to events which have relatively strong evidence from previous studies (peak Ct values, the time of peak, etc), which is how we justify using semi-informative priors. Alternative choices for the individual-level priors and the correlation between the individual-level parameters are analysed and discussed in the *Sensitivity analysis with alternative model fits* section Furthermore, we show precisely how informative the priors are by plotting the priors vs the posteriors at the covariate level (**Figures S23**) and including the individual-level priors on the individual-level posterior plots (**Fig B4**). Lastly, we justify the inclusion of semi-informative parameters within the leave-one-out model selection section, in which we show that the predictive performance of the model with semi-informative priors is higher than the model with non-informative priors.

For clarity, we present the explicit form of the upwards trajectory component (when $t_e < t \leq$

$t_e + t_p$) of the viral kinetic model for a specific example individual. We assume the individual was infected with Omicron-BA.2, that they were asymptomatic, that they are in the 20—34 years old age group and that they had 4 exposures overall. This gives the explicit equation

$$g(t;\boldsymbol{\theta}) = \frac{\left(t - \exp(\boldsymbol{\beta}_{t_e} t_e + \eta_{t_e})\right)\left(\text{logit}^{-1}(\boldsymbol{\beta}_{c_p} c_p + \eta_{c_p}) - (\boldsymbol{\beta}_{c_e} c_e + \eta_{c_e})\right)}{\exp(\boldsymbol{\beta}_{t_p} t_{p} + \eta_{t_e})} + (\boldsymbol{\beta}_{c_e} c_e + \eta_{c_e})$$

for the upwards trajectory of the viral kinetic model. For the i'th process parameter, the vector $\boldsymbol{\beta}_i$ is given by

$$\boldsymbol{\beta}_i = (\beta^{\text{BA.2}}, \ \beta^{S^-}, \beta^{20-34}, \ \beta^{4\text{exp}}, \beta^{\text{t\_last\_exp}}).$$

Each component of the $\boldsymbol{\beta}$ vector represents the value this individual's process parameters are adjusted by relative to individuals in the baseline category. For completeness, the categories of regressors used in the main analysis are VOC, symptom status, age group, number of exposures and the time-since-last-exposure, which is the same as the order written out in the example $\boldsymbol{\beta}$ vector above. The trajectory generated by $g(t;\boldsymbol{\theta})$ is then used as the mean of the Normally distributed viral kinetic likelihood function terms, with and without censoring.

Individual-level variation

We model residual individual variation across process model parameters (η_p^{ind}, where $p = t_e, t_p, t_{\text{lod}}, c_e, c_p$) as potentially correlated (i.e. an individual with a higher peak Ct value may also be more (or less) likely to have a higher Ct value at the limit of detection than an individual with a lower peak Ct value) using a multivariate normal distribution,

$$\eta_p^{\text{ind}} \sim \text{MVN}(0, \boldsymbol{\Sigma}),$$

where Σ is a 5 x 5 covariance matrix which we decompose for computational stability into a diagonal matrix containing process parameter specific scale parameters (Δ) and a symmetric correlation matrix (Ω) as follows,

$$\boldsymbol{\Sigma} = \boldsymbol{\Delta}\,\boldsymbol{\Omega}\,\boldsymbol{\Delta}$$

$$\boldsymbol{\Delta} = \begin{pmatrix} \sigma_{t_e} & 0 & 0 & 0 & 0 \\ 0 & \sigma_{t_p} & 0 & 0 & 0 \\ 0 & 0 & \sigma_{t_{lod}} & 0 & 0 \\ 0 & 0 & 0 & \sigma_{c_e} & 0 \\ 0 & 0 & 0 & 0 & \sigma_{c_p} \end{pmatrix}$$

$$\mathbf{\Omega} = \begin{pmatrix} 1 & \omega_{t_e, t_p} & \omega_{t_e, t_{lod}} & \omega_{t_e, c_e} & \omega_{t_e, c_p} \\ \omega_{t_p, t_e} & 1 & \omega_{t_p, t_{lod}} & \omega_{t_p, c_e} & \omega_{t_p, c_p} \\ \omega_{t_{lod}, t_e} & \omega_{t_{lod}, t_p} & 1 & \omega_{t_{lod}, c_e} & \omega_{t_{lod}, c_p} \\ \omega_{c_e, t_e} & \omega_{c_e, t_p} & \omega_{c_e, t_{lod}} & 1 & \omega_{c_e, c_p} \\ \omega_{c_p, t_e} & \omega_{c_p, t_p} & \omega_{c_p, t_{lod}} & \omega_{c_p, c_e} & 1 \end{pmatrix}$$

where ω_{p_1, p_2} dictates the correlation between the process parameters p_1 and p_2. We use a Lewandowski-Kurowicka-Joe (LKJ) prior for the symmetric correlation matrix $\mathbf{\Omega}$ and a weakly informative half-normal prior for the scale parameters

$$\mathbf{\Omega} \sim \text{LKJCorr}(\upsilon)$$
$$\sigma_p \sim \text{Half-Normal}(0, 0.2).$$

For the LKJ prior $\upsilon = 1$ results in a uniform prior over all correlations, $\upsilon < 1$ places more weight on larger correlations and $\upsilon > 1$ places more weight on small amounts of correlations. By default, we set $\upsilon = 1$. On top of this potentially correlated process parameter model, we also model the extreme scenario where we assume no correlation between process parameters. I.e. $\omega_{p_1, p_2} = 0$ for all parameter combinations.

As the multivariate normal density and LKJ prior on correlation matrices both require their matrix parameters to be factored we have parameterised our model directly in terms of Cholesky factors of correlation matrices using the multivariate version of the non-centered parameterisation, as recommended by the Stan User Manual. This increases numerical stability and sampling efficiency, reducing the computational requirements of model fitting.

Bayesian linear regression structure

To stratify by the key covariates included in our dataset and often present in other similar datasets, we include a linear regression structure within our modelling framework. In the model structure section, these are the β terms multiplying the individual-level parameters. We then use the posterior distributions for these parameters to adjust our population-level posteriors to measure the relative and absolute effect size for each covariate we regress against (**Fig 3, Fig 4**).

Gene target and swab type adjustment

Using the same programmatic structure as the linear regression used to regress against the covariates of interest, we adjusted for which gene targets were detected and which swab type

was used (wet or dry).

Individual-level posterior and posterior predictive distributions

The parameters of the viral kinetic part of the model were fit for each individual. Using these estimated individual-level parameters, we are able to simulate likely Ct trajectories and check the goodness-of-fit of the model against the observed Ct value data. We plot the three timing posterior distributions (**Fig F, S7 and S8**), the Ct peak value posterior distributions (**Fig I**) and the resulting individual-level Ct value trajectories all stratified by VOC (**Fig J, S11 and S12**) with the underlying data we fit to: symptom onset and Ct values at the three gene targets investigated overlaid on the fits. **Figs M, N** and **O** show the same posterior distributions as **Figs F, G** and **H**, with the individual-level priors included for comparison.

Computational details

We implemented the model using R 4.2.2 (6) and CmdStan 2.3.1 (7). We ran the model for 3000 iterations, after 1000 warm-up iterations. We used the standard R-hat < 1.05 diagnostic to assess convergence of the chains for each model parameter. The model fit took no longer than a few minutes on a 2021 M1 MacBook Pro, passed all the in-built standard diagnostic tests and fit with no divergent transitions.

Timing parameters

Delta-infected individuals

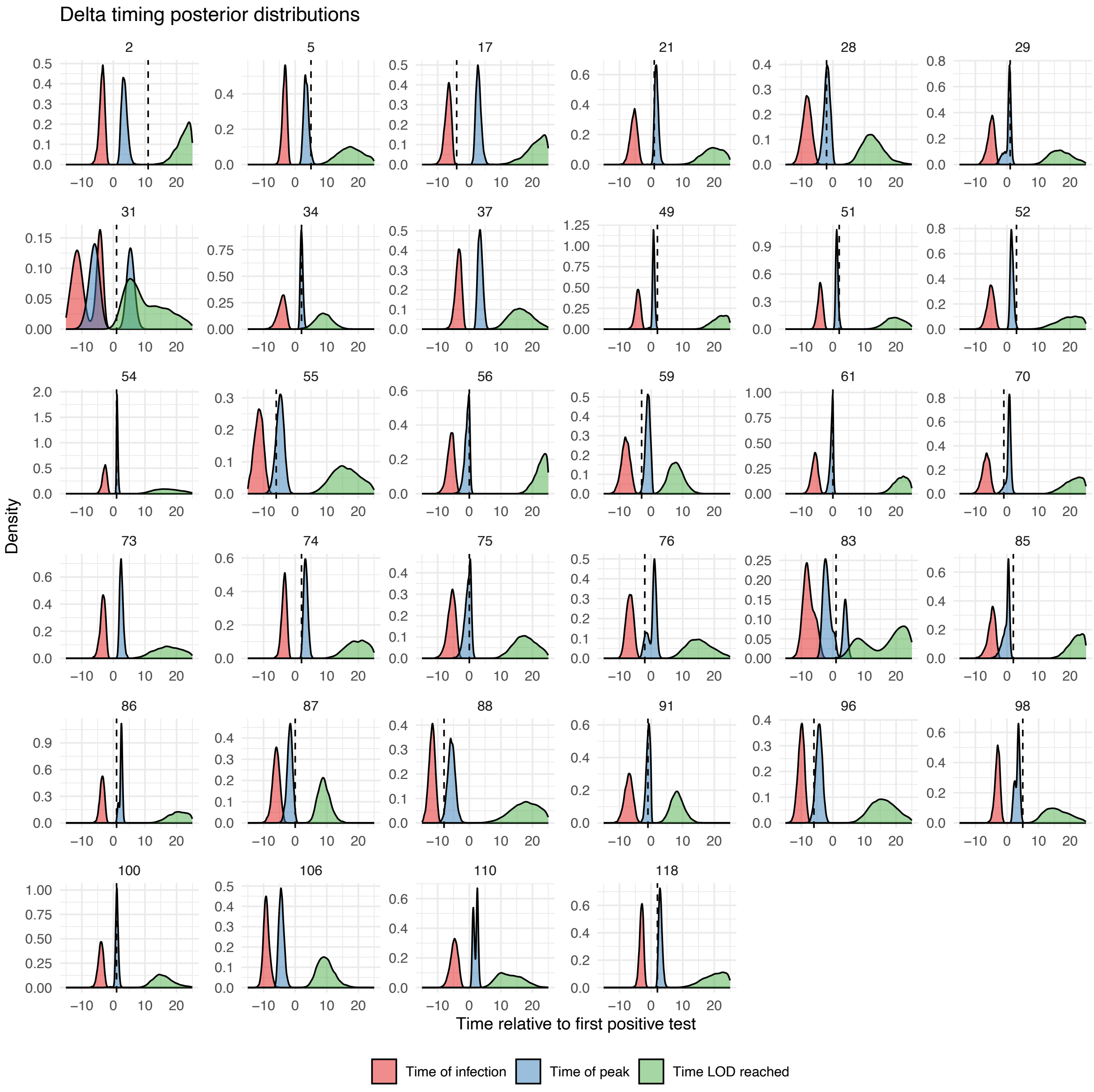

Fig F. Posterior distributions for the individual-level time of infection, time at which the peak Ct value is reached and the time the LOD is reached for individuals infected with the Delta variant. The IDs match the other individual-level posterior plots (**Fig F-O**). All times are relative to each individual's first positive test. Dashed vertical lines represent the time at which symptoms began for each individual, where reported.

Omicron (BA.1)-infected individuals

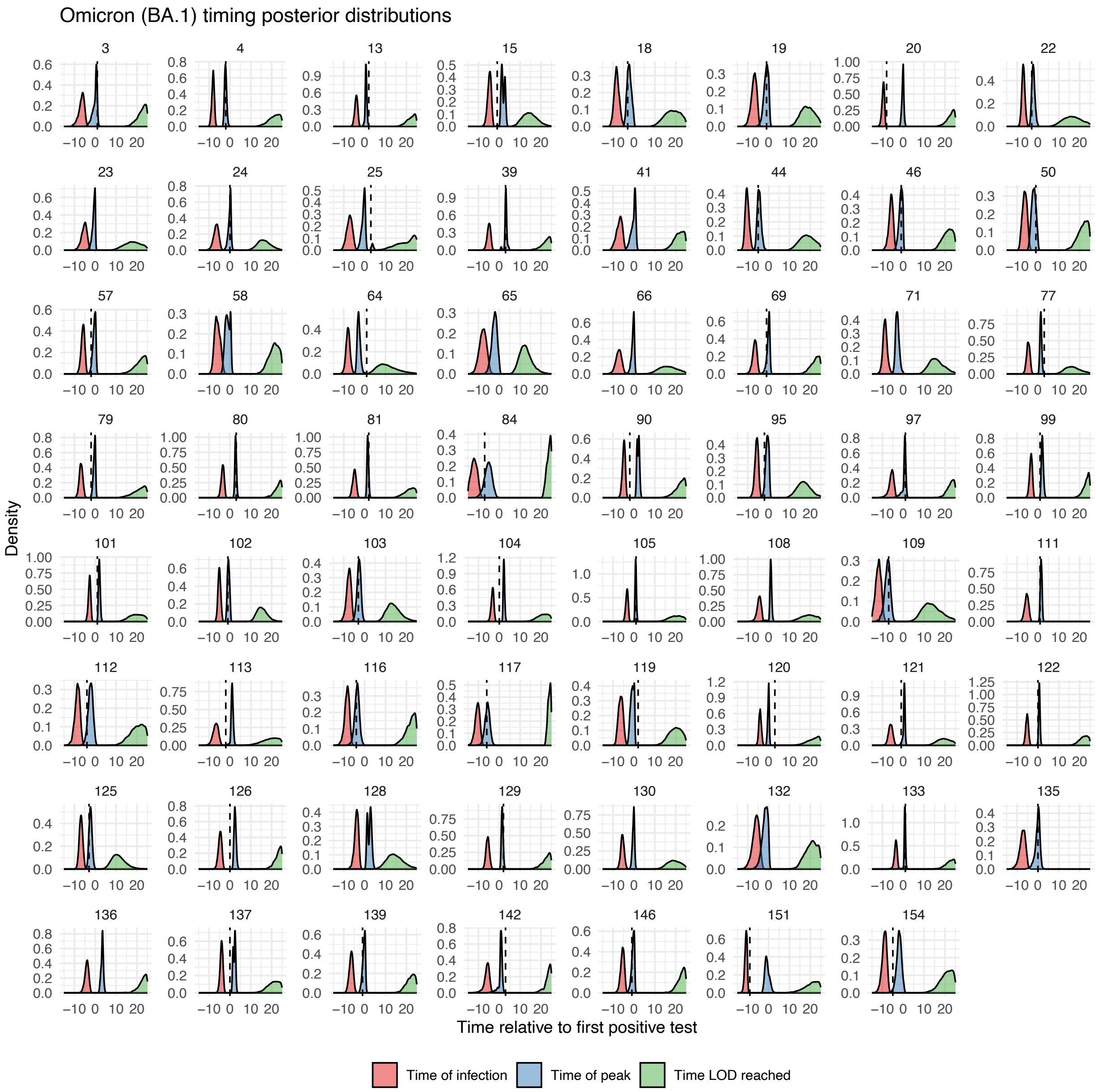

Fig G. Posterior distributions for the individual-level time of infection, time at which the peak Ct value is reached and the time the LOD is reached for individuals infected with the Omicron BA.1 variant. The IDs match the other individual-level posterior plots (**Fig F-O**). All times are relative to each individual's first positive test. Dashed vertical lines represent the time at which symptoms began for each individual, where reported.

Omicron (BA.2)-infected individuals

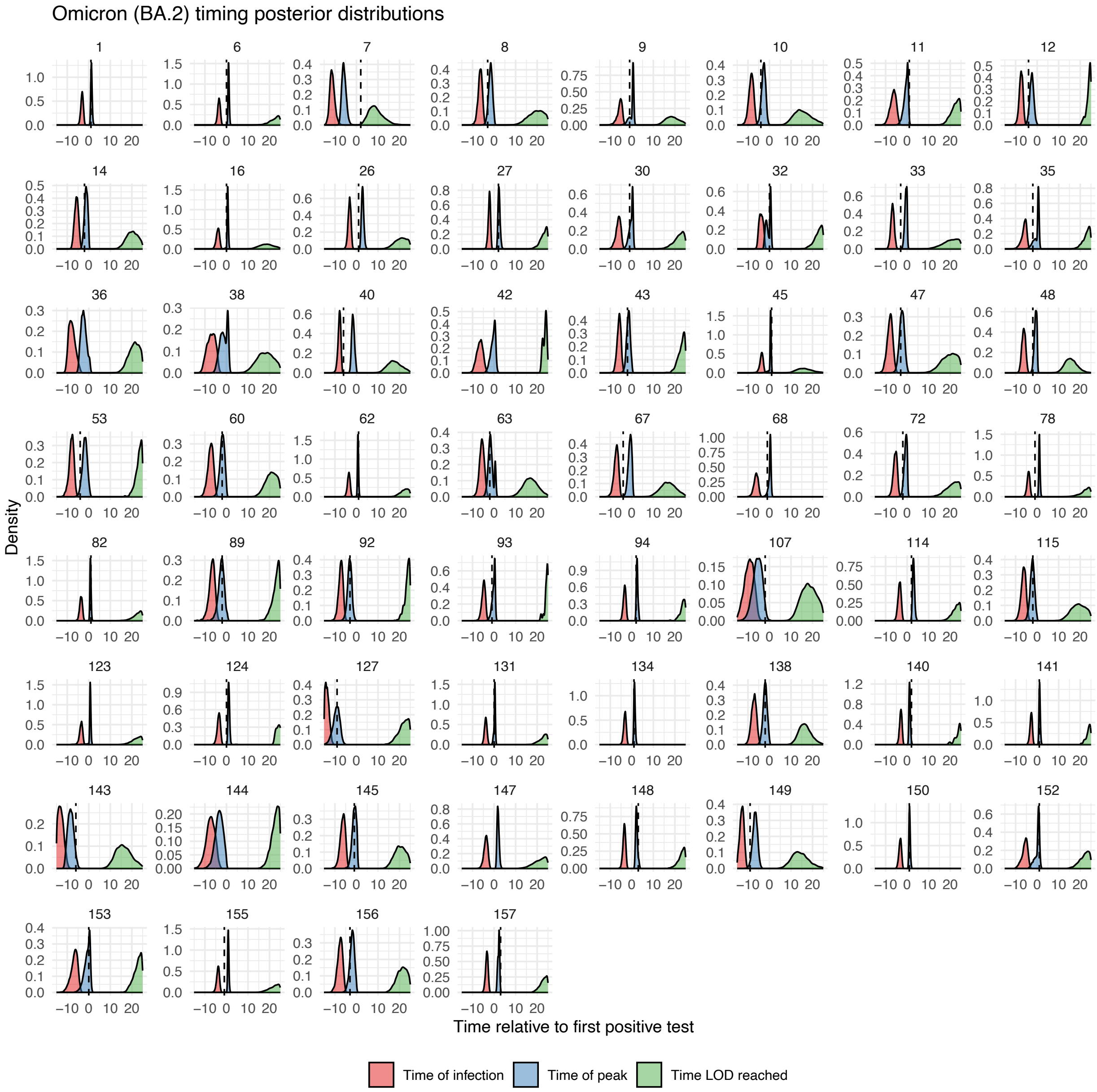

Fig H. Posterior distributions for the individual-level time of infection, time at which the peak Ct value is reached and the time the LOD is reached for individuals infected with the Omicron BA.2 variant. The IDs match the other individual-level posterior plots (**Fig F-O**). All times are relative to each individual’s first positive test. Dashed vertical lines represent the time at which symptoms began for each individual, where reported.

Ct value parameters

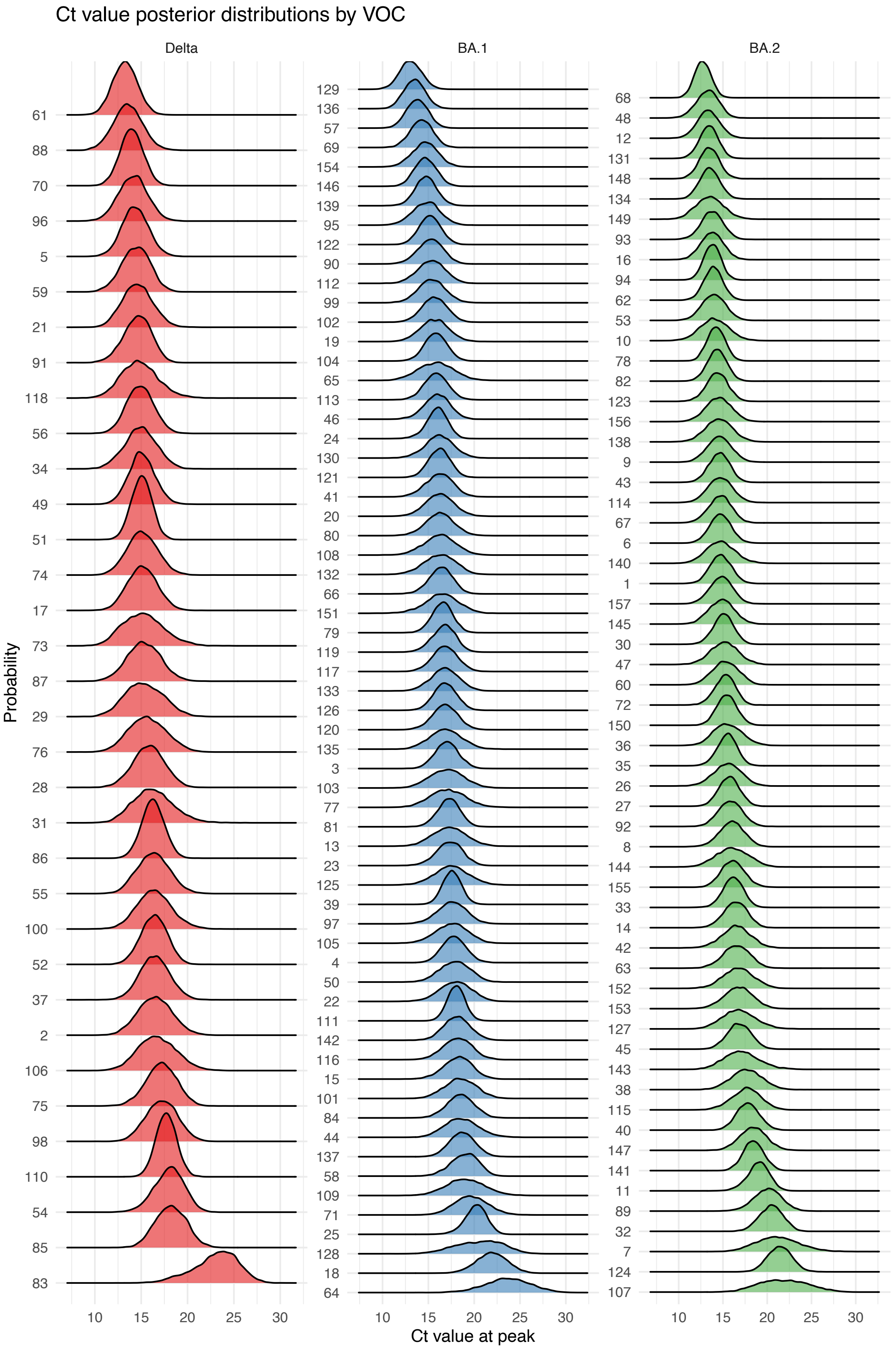

Fig I. Posterior distributions for the individual-level peak Ct value, with IDs which match the other individual-level posterior plots (**Fig F-O)**.

Viral kinetics model fits

We present the fitted Ct trajectories stratified by the three VOCs under investigation. The fitted trajectories presented are generated using the fitted individual-level parameters for each individual.

Delta-infected individuals

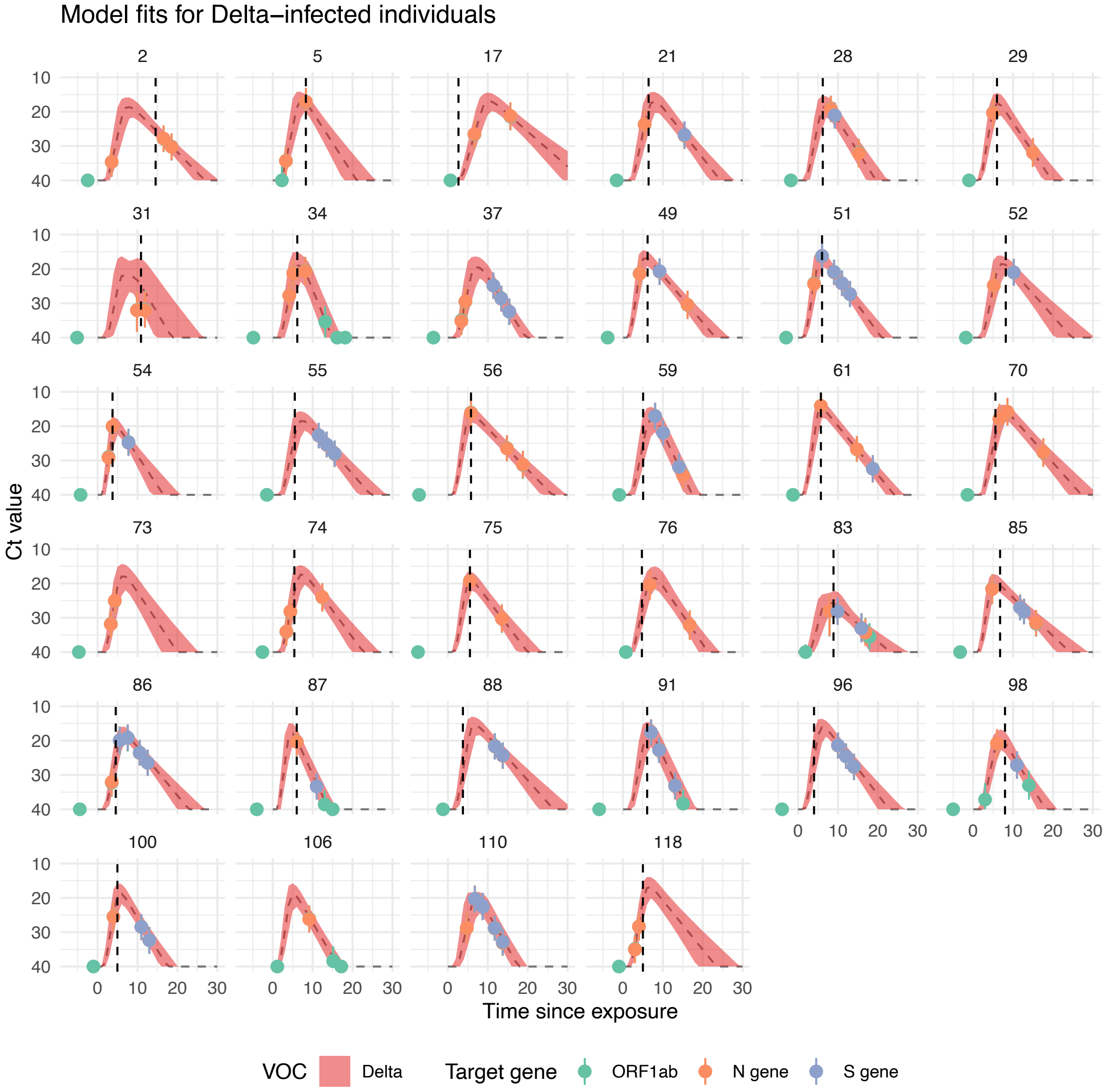

Fig J. Posterior predictive distributions for all Delta-infected individuals, produced by simulating the Ct trajectory model using the inferred posterior distributions shown in **Fig F-O**, with matching IDs. All times are relative to each individual's estimated time of exposure. Dashed vertical lines represent the time at which symptoms began for each individual, where reported.

Omicron (BA.1)-infected individuals

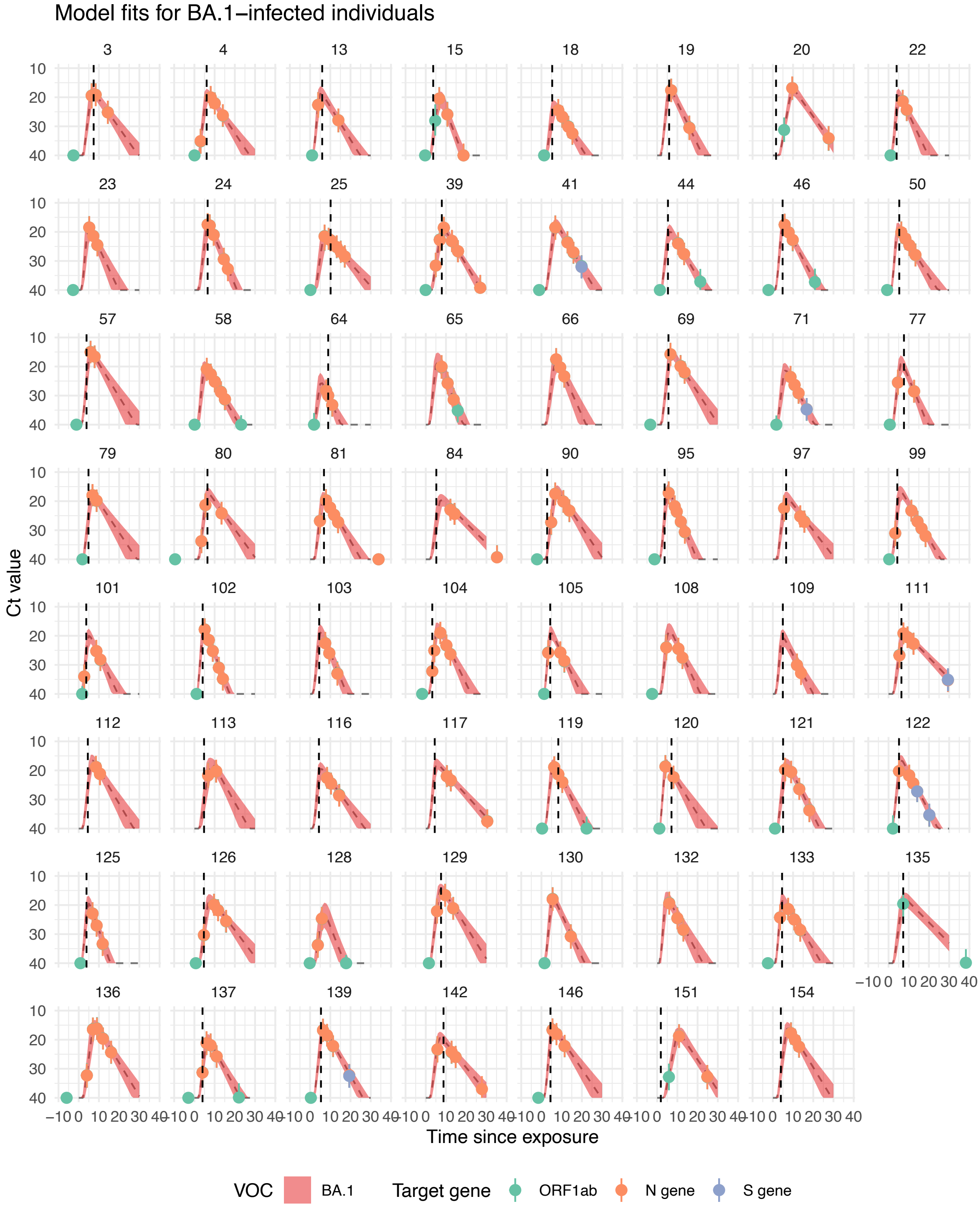

Fig K. Posterior predictive distributions for all Omicron (BA.1)-infected individuals, produced by simulating the Ct trajectory model using the inferred posterior distributions shown in **Fig F-O**, with matching IDs. All times are relative to each individual's estimated time of exposure. Dashed vertical lines represent the time at which symptoms began for each individual, where reported.

Omicron (BA.2)-infected individuals

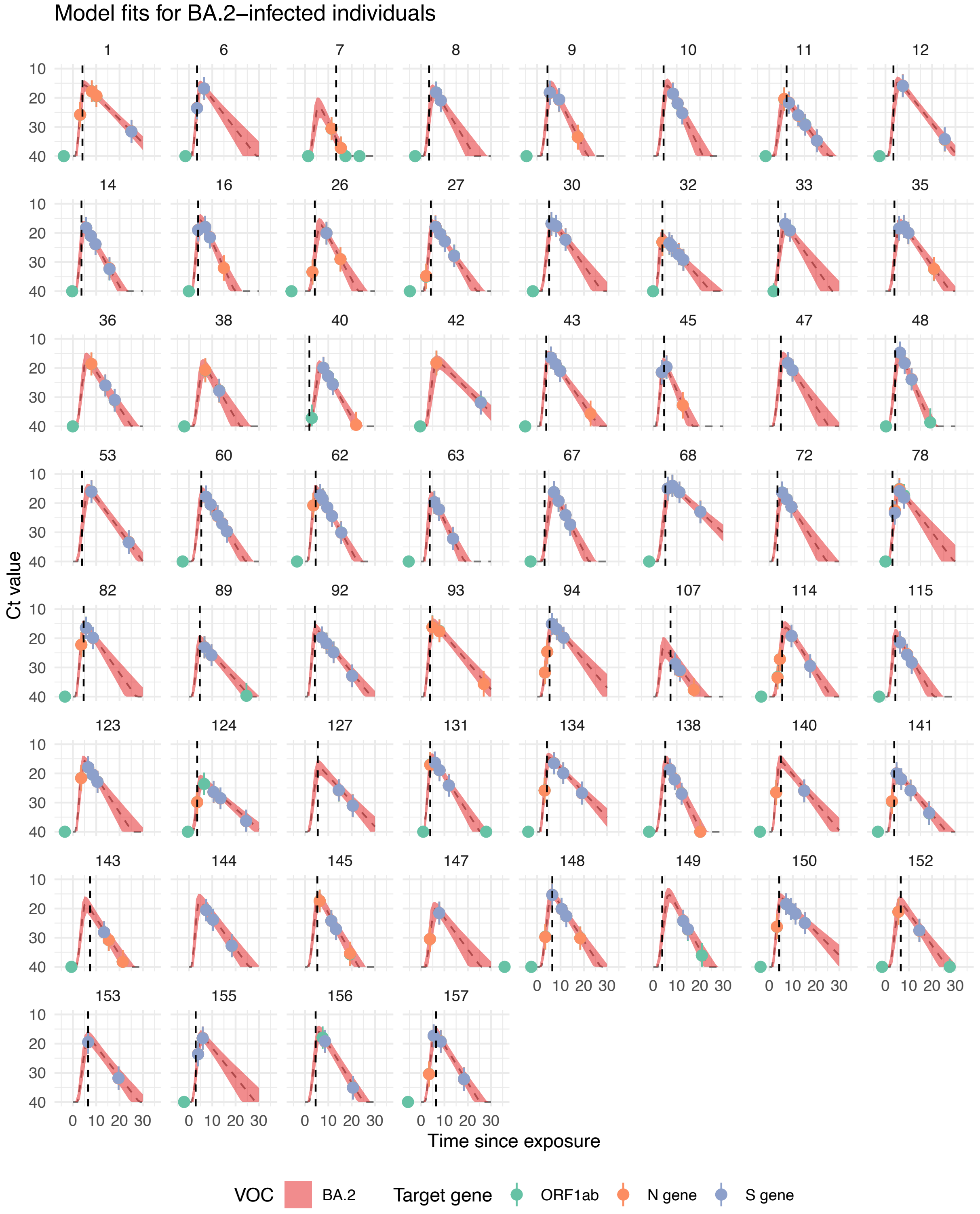

Fig L. Posterior predictive distributions for all Omicron (BA.2)-infected individuals, produced by simulating the Ct trajectory model using the inferred posterior distributions shown in **Fig F-O**, with matching IDs. All times are relative to each individual's estimated time of exposure. Dashed vertical lines represent the time at which symptoms began for each individual, where reported.

Timing parameters, prior vs posterior

Delta-infected individuals

In a similar style to the individual-level posterior distribution plots (**Fig F-J**), we plot the individual-level prior distributions on the same plot as the posterior distributions, to indicate the overall effect our semi-informative priors have on our overall posterior distributions. As can be seen from the new figures (**Fig M-O**), the posterior distributions are well-informed relative to the semi-informative priors and where the data influences them, away from the values with high density within the priors.

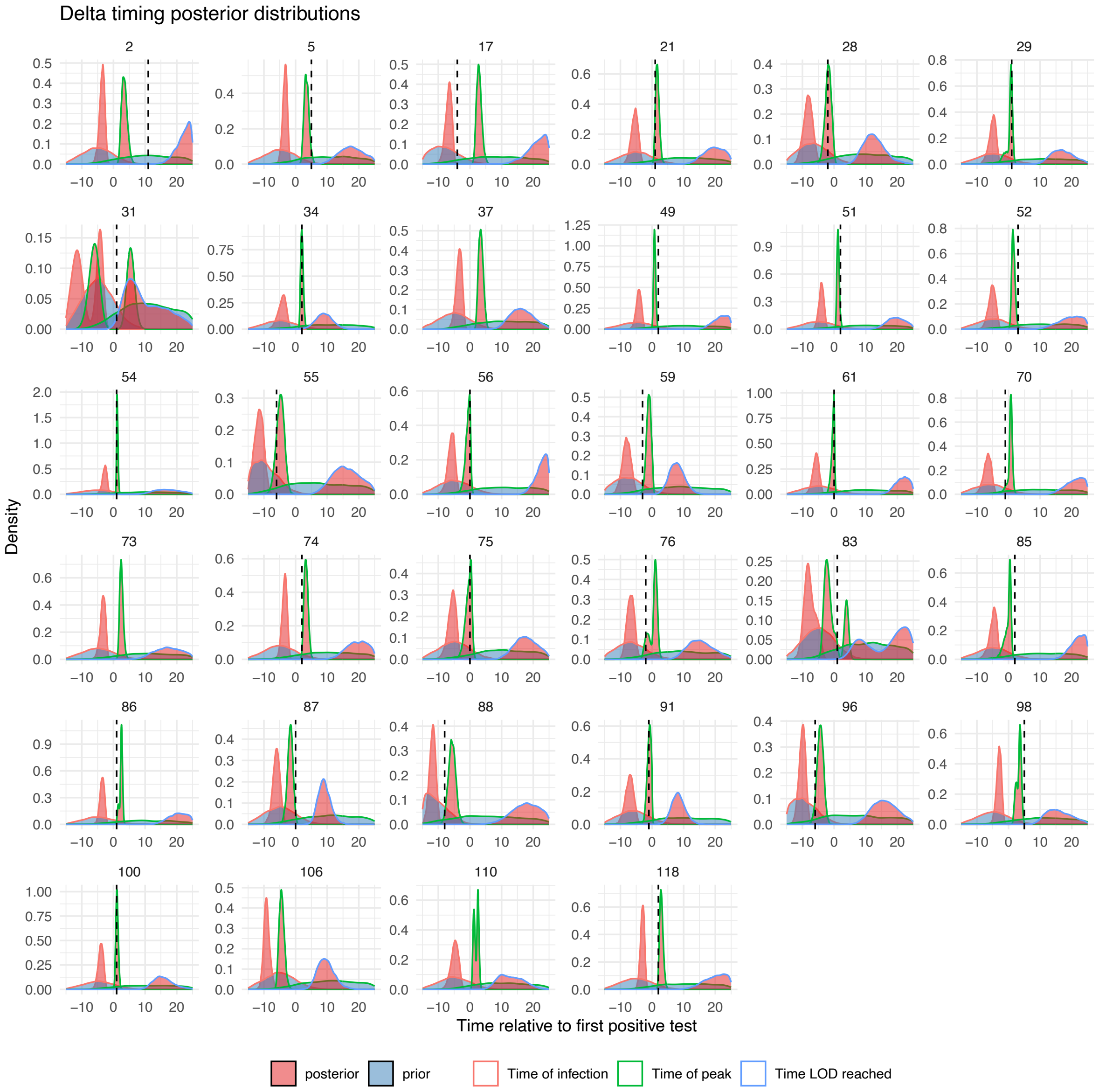

Fig M. Timing posteriors for Delta-infected individuals, with individual-level priors. Same as **Fig F**, with the individual-level priors included.

Omicron (BA.1)-infected individuals

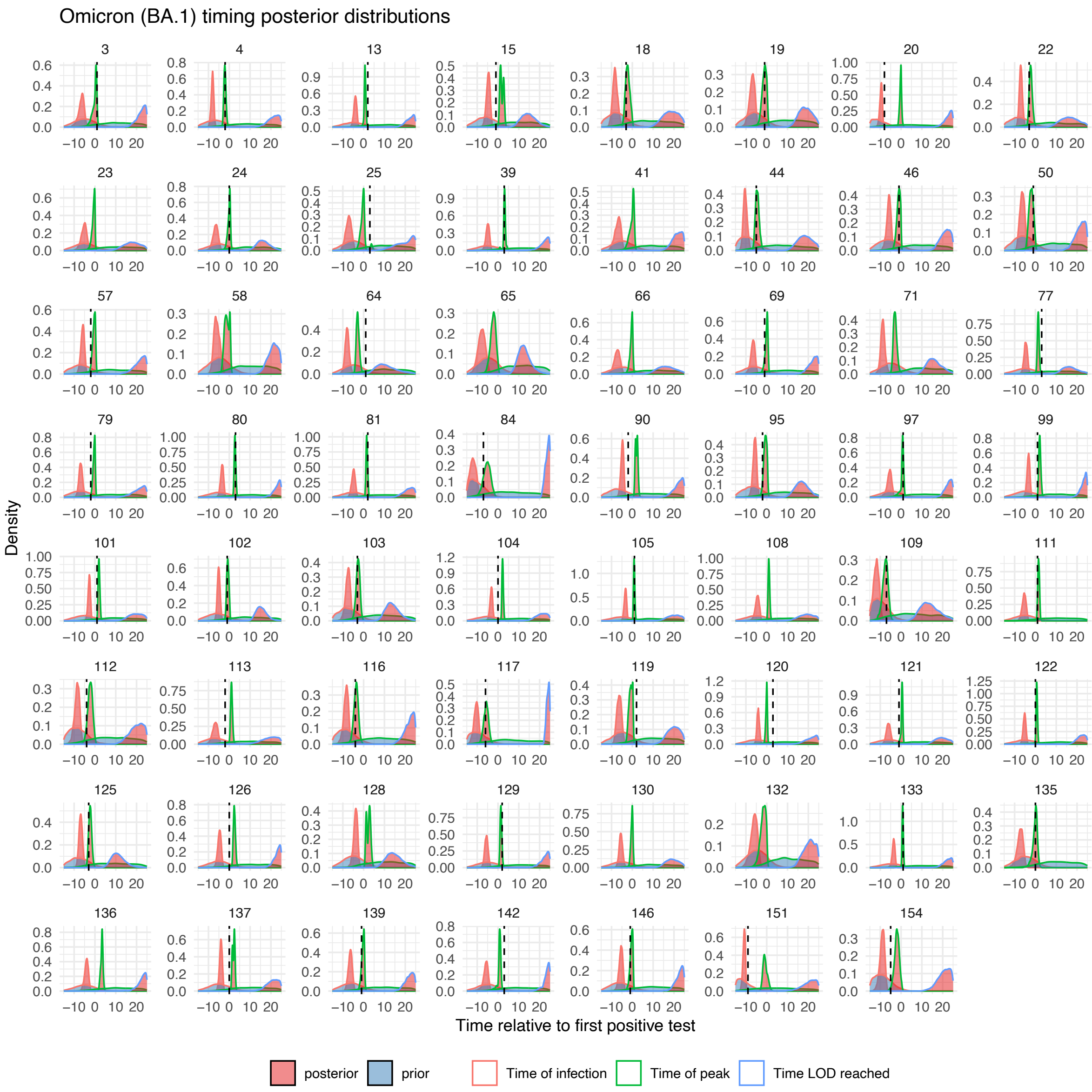

Fig N. Timing posteriors for Omicron (BA.1)-infected individuals, with individual- level priors. Same as **Fig G** , with the individual-level priors included.

Omicron (BA.2)-infected individuals

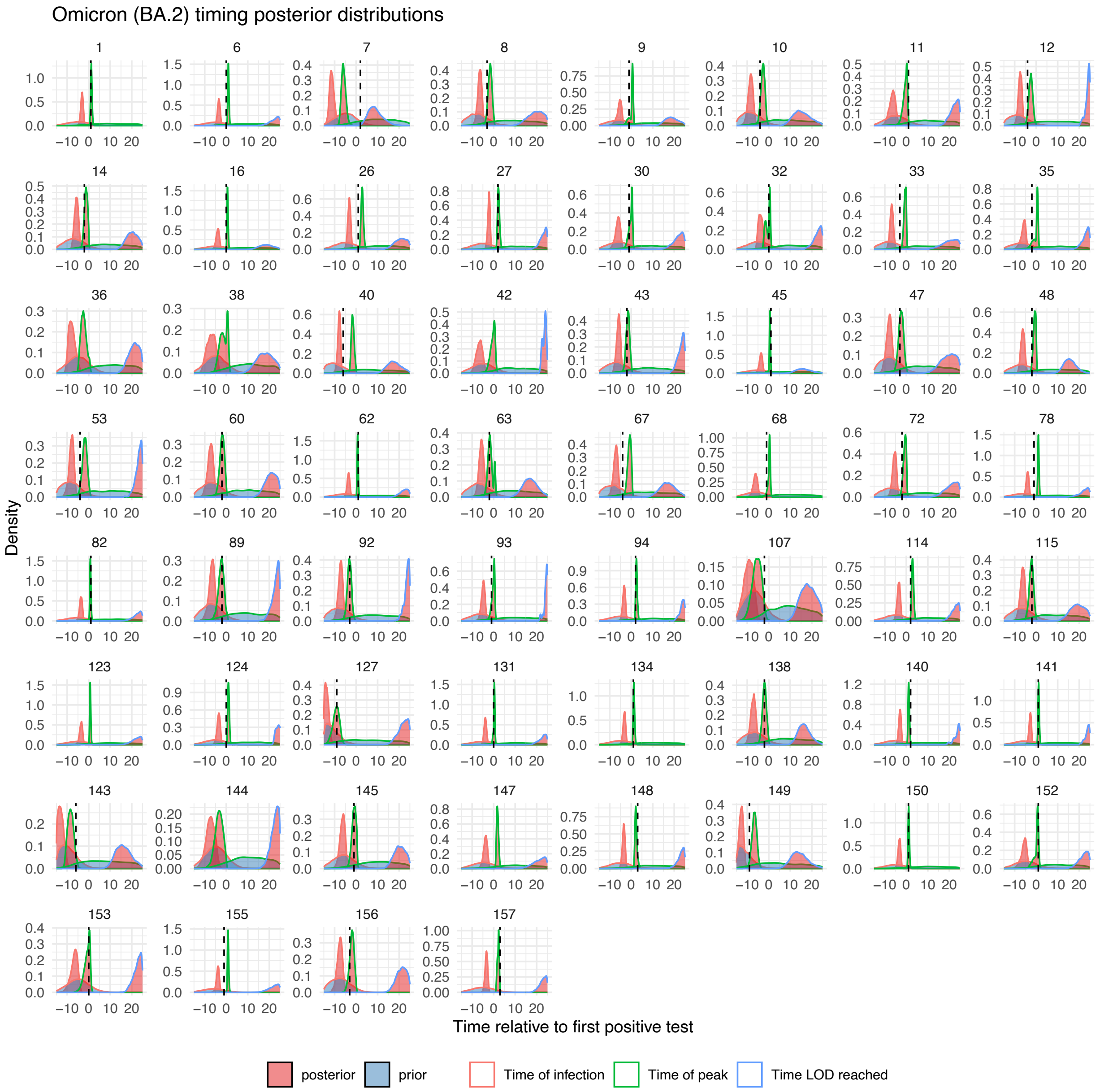

Fig O. Timing posteriors for Omicron (BA.2)-infected individuals, with individual- level priors. Same as **Fig H**, with the individual-level priors included.

Timing of rapid test positivity

For rapid test positivity (assumed Ct = 30), baseline trajectories crossed the positivity threshold at 3.7 (95% CrI: 2.8—4.4) days after exposure and would have remained positive until 17.9 (95% CrI: 15.6—19.6) days after exposure. We estimated that Delta trajectories would turn rapid test positive at an almost identical time to the baseline trajectories (for symptomatic BA.1 infections in 34-49yo with 4 previous exposures) but would only remain positive until 16.7 (95% CrI: 141—18.8) days after exposure. We estimated that BA.2 trajectories would turn positive 3.1 (95% CrI: 2.2—3.9) days after exposure and would remain positive until 18.7 (95% CrI: 16.2—20.7) days after exposure. Trajectories for all other covariates would turn positive at almost identical times to the baseline trajectories. However, we estimated substantial variation in the duration of rapid test positivity for the other covariate categories. Trajectories would become rapid test negative for participants with 3 or 5+ post exposures sooner than baseline individuals; specifically at 15.7 (95% CrI: 11.1—15.7) and 14.2 (95% CrI: 10.4—13.9) respectively. Lastly, we found that trajectories for participants in the 50+ age group would become negative later than for reference individuals, at 19.3 (95% CrI: 16.4—21.8) days after exposure.

Tables of population-level posterior estimates

S3 Table. Table of the median and 95% credible intervals of the population-level Ct model parameters by VOC.

S4 Table. Table of the median and 95% credible intervals of the population-level Ct model parameters by symptom status.

S5 Table. Table of the median and 95% credible intervals of the population-level Ct model parameters by total number of antigenic exposures.

S6 Table. Table of the median and 95% credible intervals of the population-level Ct model parameters by age.

Sensitivity analysis with alternative model fits

To check the sensitivity of the statistical and viral kinetic components of our model to different covariates and prior distributions, we ran a number of model variations. Specifically, we re-fit our model considering four different changes to model structure in turn: omission of all covariates other than VOC; increasing the potential for variation to be attributed to individual-level effects; removing the estimation of correlation structure between individual- level responses and removing symptom onset data from the likelihood.

No covariates other than VOC

When the model was fitted with only VOC as a covariate, we observed similar trends in effect size across VOCs, but with substantially different and more varied point estimates for Ct value at peak and narrower credible intervals. This reflects our finding that additional covariates are likely to influence viral dynamics – such as prior exposures that would have generated immunity – and omission of these covariates can result in too much of the individual-level variation being attributed to the VOC rather than shifting population characteristics over time.

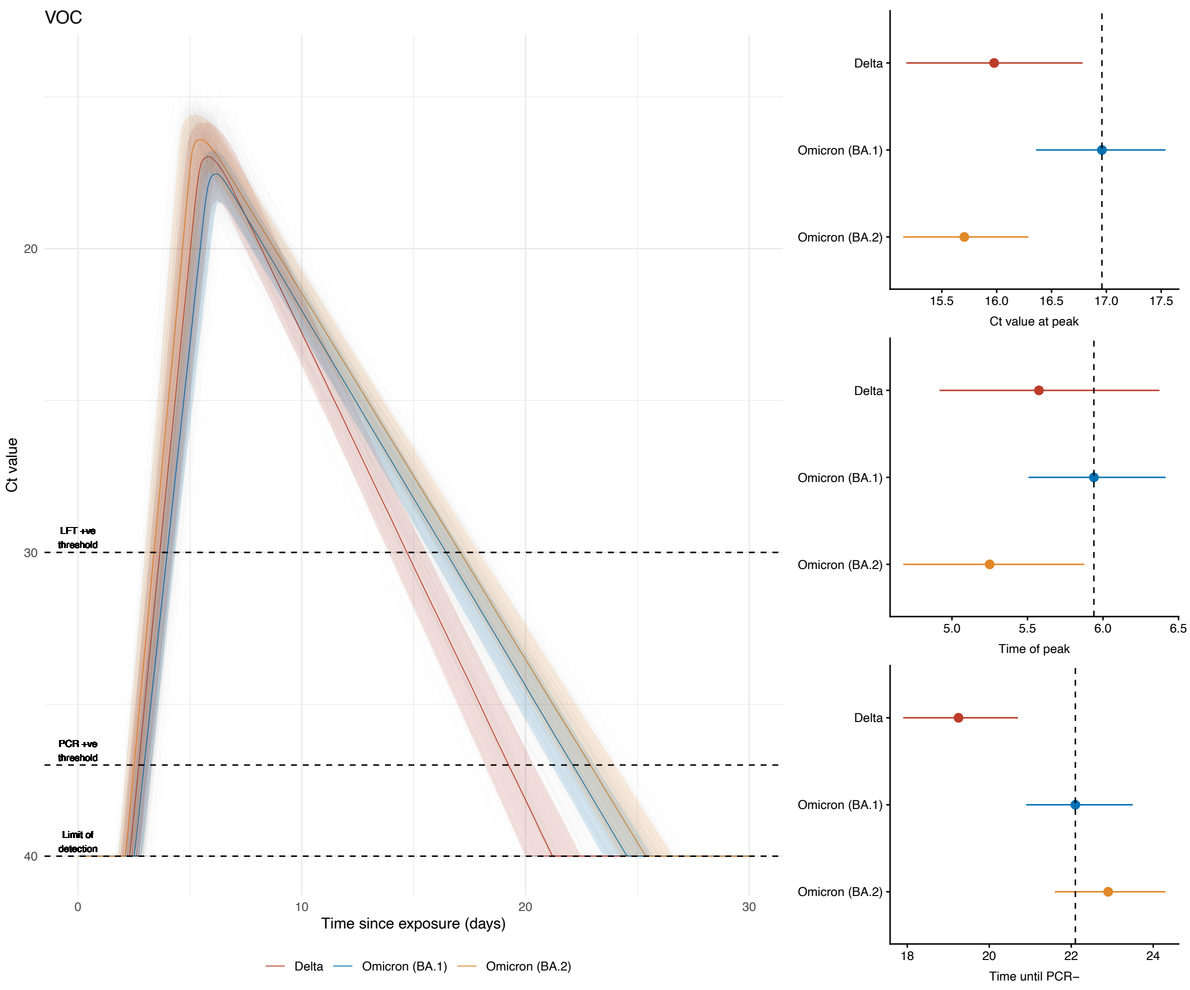

Fig P. Population-level fit for the model when only the infecting VOC was used as a covariate.

Greater individual-level variation

When we changed the prior distribution assumptions to allow for greater individual-level variation in response, our estimates had larger uncertainty, but similar findings with regards to the effect of prior exposures and age, indicating that these population-level characteristics still had a discernible effect against a background of noisy individual-level responses.

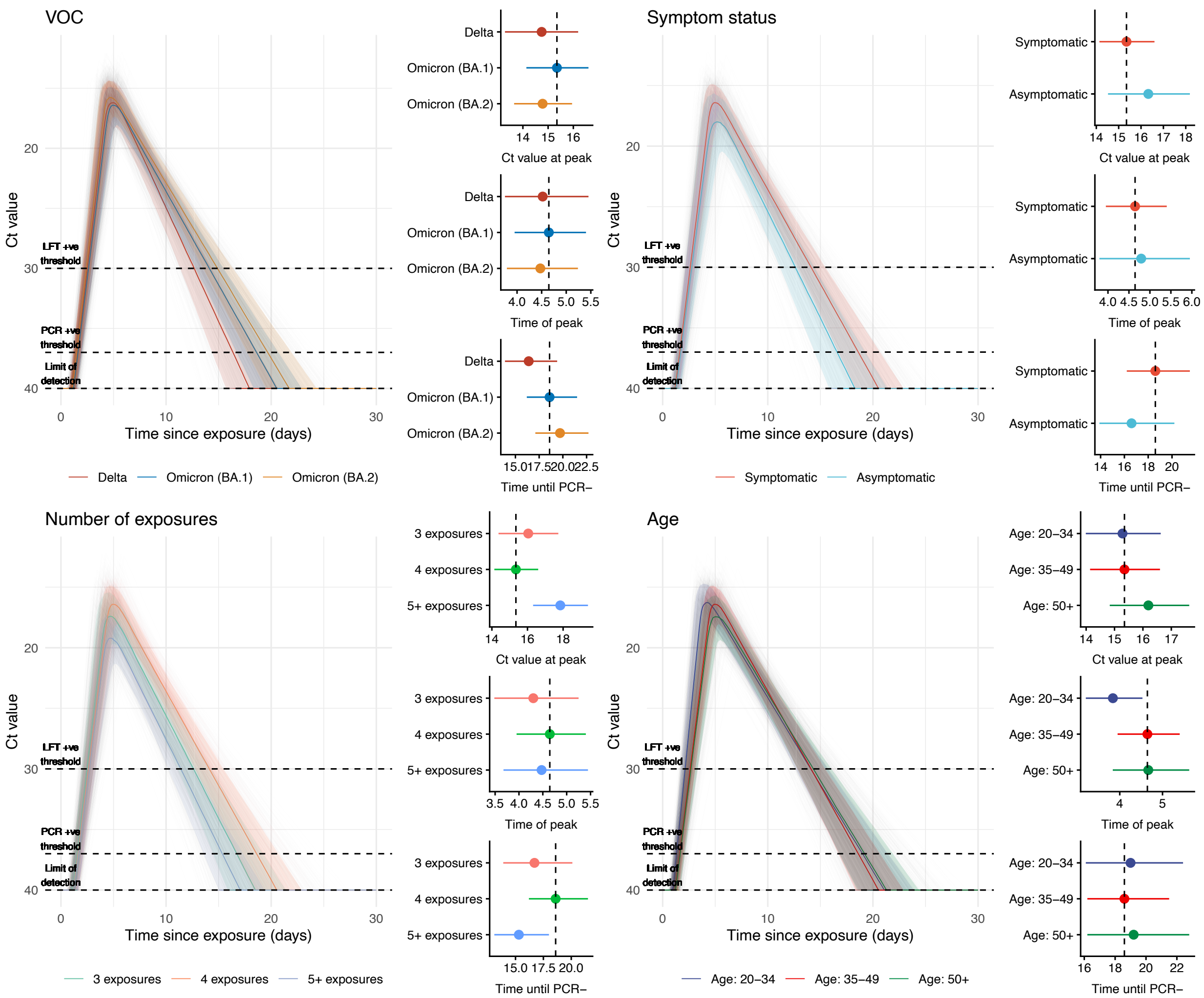

Fig Q. Population-level fits for the model when substantially higher levels of individual- level variation were permitted.

No correlation between individual-level parameters

To assess how much individual-level parameters were jointly informed by the assumed correlation structure, we fitted the model assuming no correlation between individual-level parameters. We obtained similar estimates to our main analysis, again suggesting that population-level characteristics had a discernible effect even when individual-level dynamics were fitted with a prior representing no correlation.

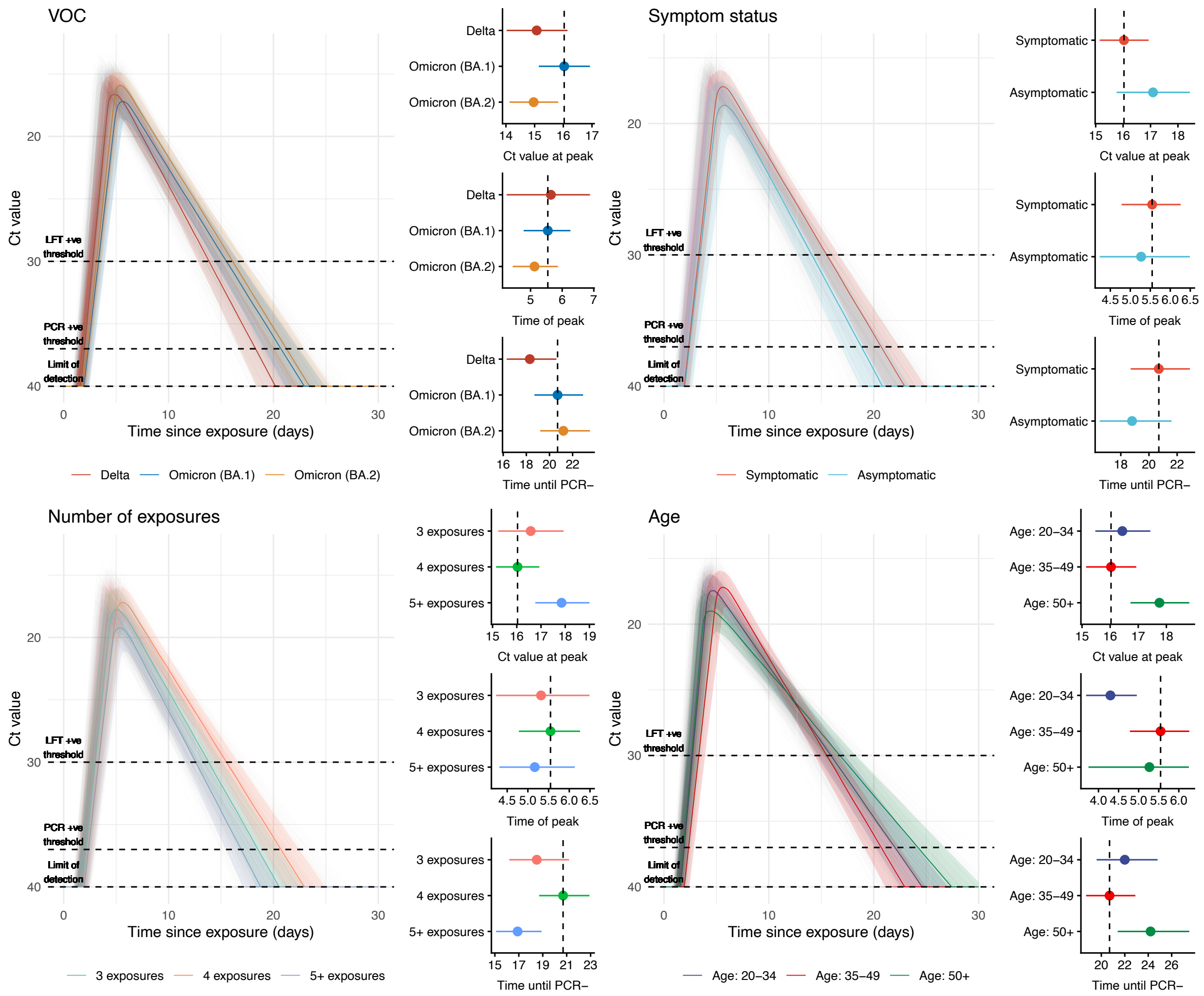

Figure S18. Population-level fits for the model when parameterised with a prior that almost entirely removes correlation between the individual-level parameters, controlled by the multivariate normal distribution structure. Specifically, the parameter of the LKJ-prior used to control the multivariate normal distribution was set to a far higher value ($\eta = 50$), whereby high values correspond to lower and lower levels of permitted correlation between individual-level parameters.

No symptom onset data used in likelihood

When we omitted symptom onset data from the likelihood, we obtained similar conclusions about the role of prior exposures and age, but lower point estimates for the time until peak Ct value. This suggests that symptom onset data – anchored to infection time via our prior on the incubation period – is important for supplementing the sparsity in early post-infection Ct measurements and its interaction with fast-changing Ct values early in the infection.

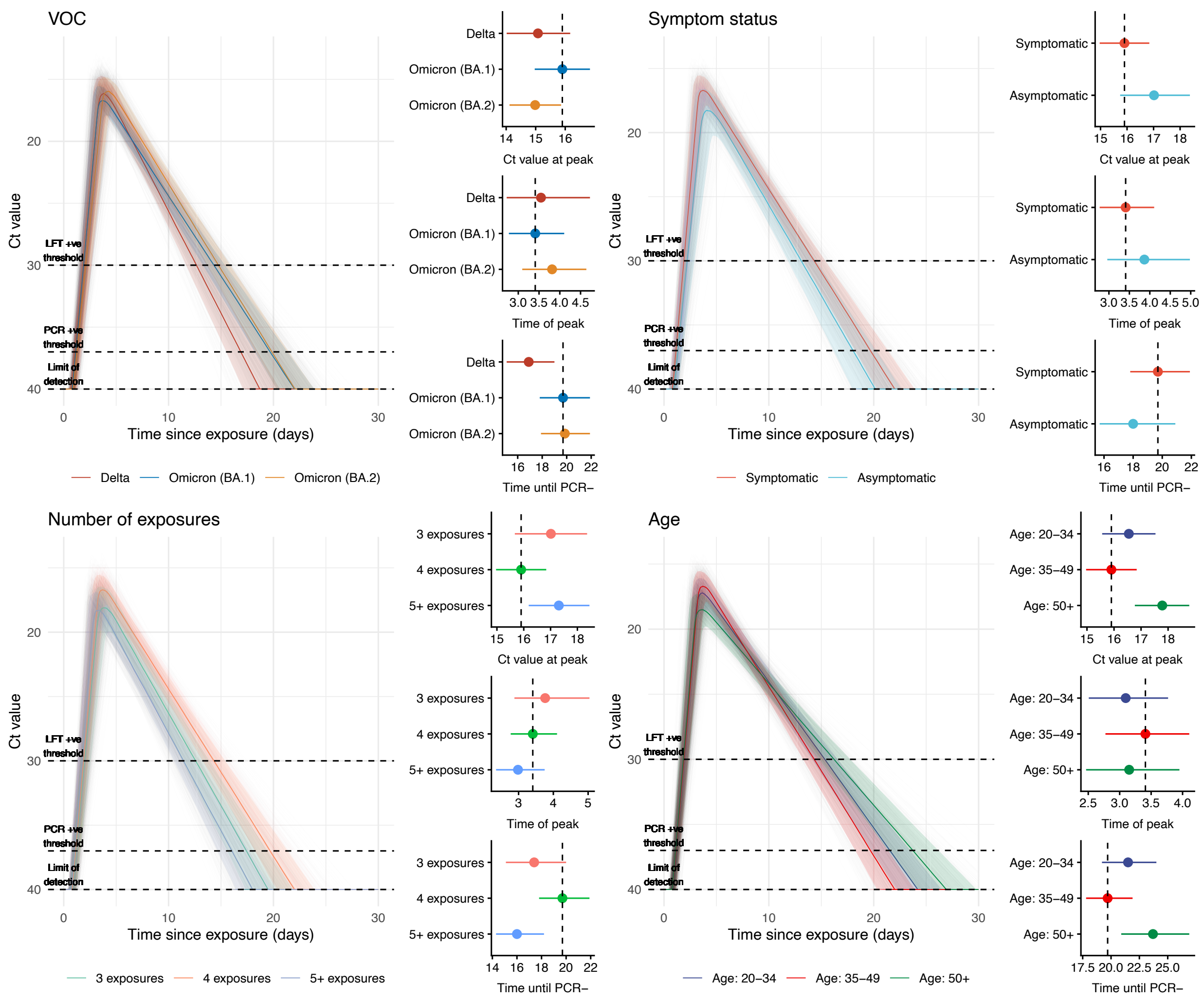

Fig S Covariate-level fits for the model when symptom onset data was omitted from the fitting. The time of exposure for each individual is informed in the model presented in the main text by a combination of the Ct trajectory model, fit to Ct value data, and the incubation period model, fit to symptom onset data. We refit the model after removing the symptom onset component of the likelihood, arriving at the above population-level fits.

Less informative priors

To investigate how sensitive our results from our main analysis are to our semi-informative priors, we widen the covariate-level priors by increasing the variance parameter considerably for the three main process parameters: Ct value at peak, timing of the peak and timing the LOD is reached. We still used Normal distributions for all covariate-level priors, as they are highly recommended for high-dimensional hierarchical models such as this one, to aid with sampling from complex geometries. However, where the variance parameter was between 0.5 and 1 for all semi-informative priors — with mean values chosen from literature — we multiply all variance parameters by 5, widening the priors considerably. The parameters are on logit (Ct value parameters) or log scales (timing parameters), meaning that this increase in the variance when measured in natural units (Ct values or time (days)), should also be interpreted on these same scales. The results using uninformative priors are in line with the semi-informative priors (**Fig T**). The direction of all effect sizes is the same between the two and all magnitudes are within each other's credible intervals. The overall uncertainty for the uninformative prior case is slightly wider, but at a level hard to distinguish visually. We conclude from this that our results from our main analysis using semi-informative priors are not overly sensitive to the choices made and that our resulting estimates are mostly informed by our modelling framework conditioned on our dataset.

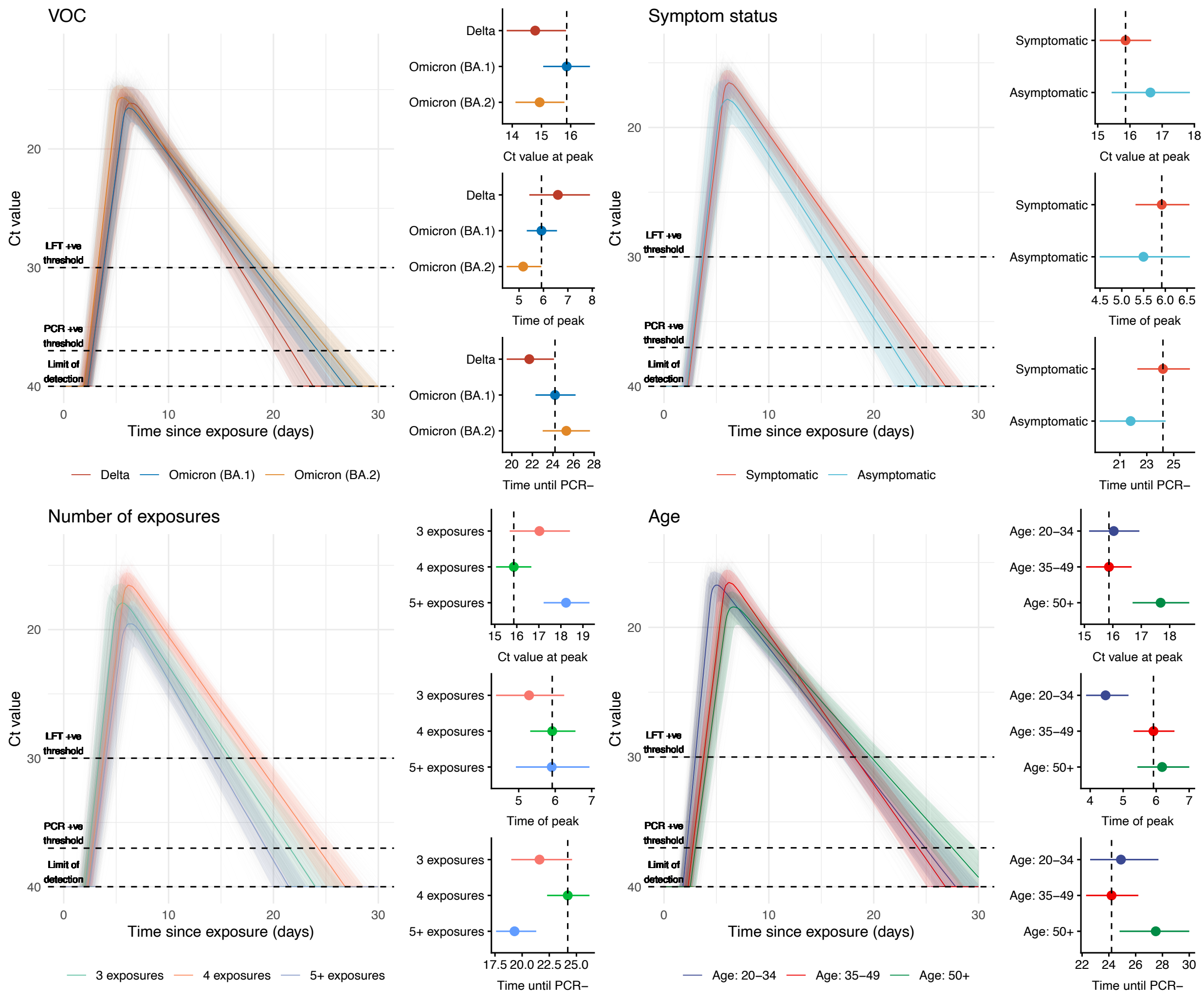

Fig T. Covariate-level model fits with less-informative priors. We refit the model with the covariate-level priors widened by a factor of 5. We see that the results are in line with those using semi-informative priors.

Alternative Ct threshold for Figure 4

To investigate the effect of the assumed Ct value threshold used in Figure 4 (Ct = 20), we varied the Ct threshold (Ct = 25) and replotted Figure 4.

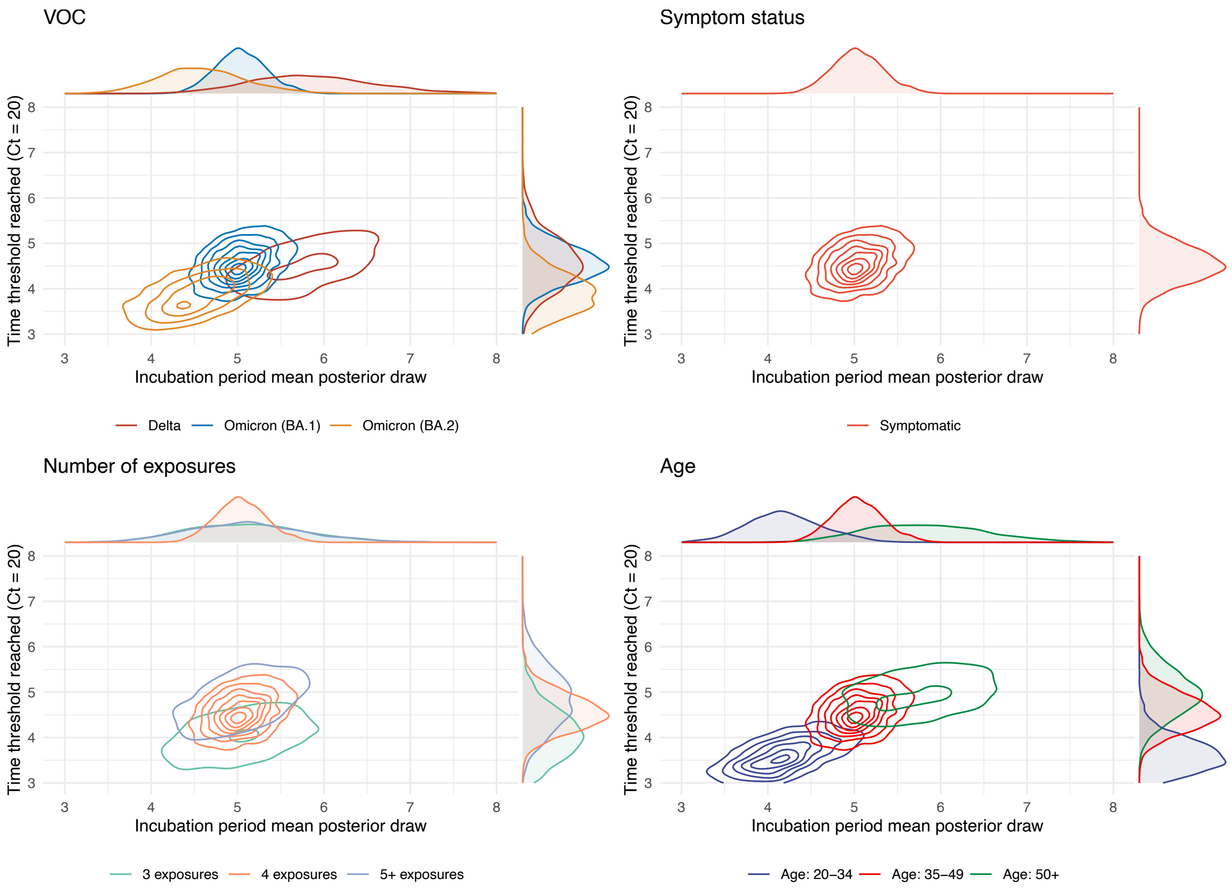

Fig U. Bivariate density plots of median incubation periods against the time at which trajectories surpassed an assumed Ct value (Ct = 25), for each covariate. All panels: We replot **Figure 4** in the main text with a different assumed Ct value threshold (Ct = 25 vs Ct = 20). **A.** Parameter values for the three VOCs considered. **B.** Parameter values corresponding to symptomatic infections. **C.** Parameter values corresponding to the numbers of exposures considered. **D.** Parameter values for the age groups considered.

Incubation period estimates

To investigate differences in the overall incubation period estimates, sampled using both the inferred mean and standard deviation parameters, are given, stratified by each covariate included in the main analysis.

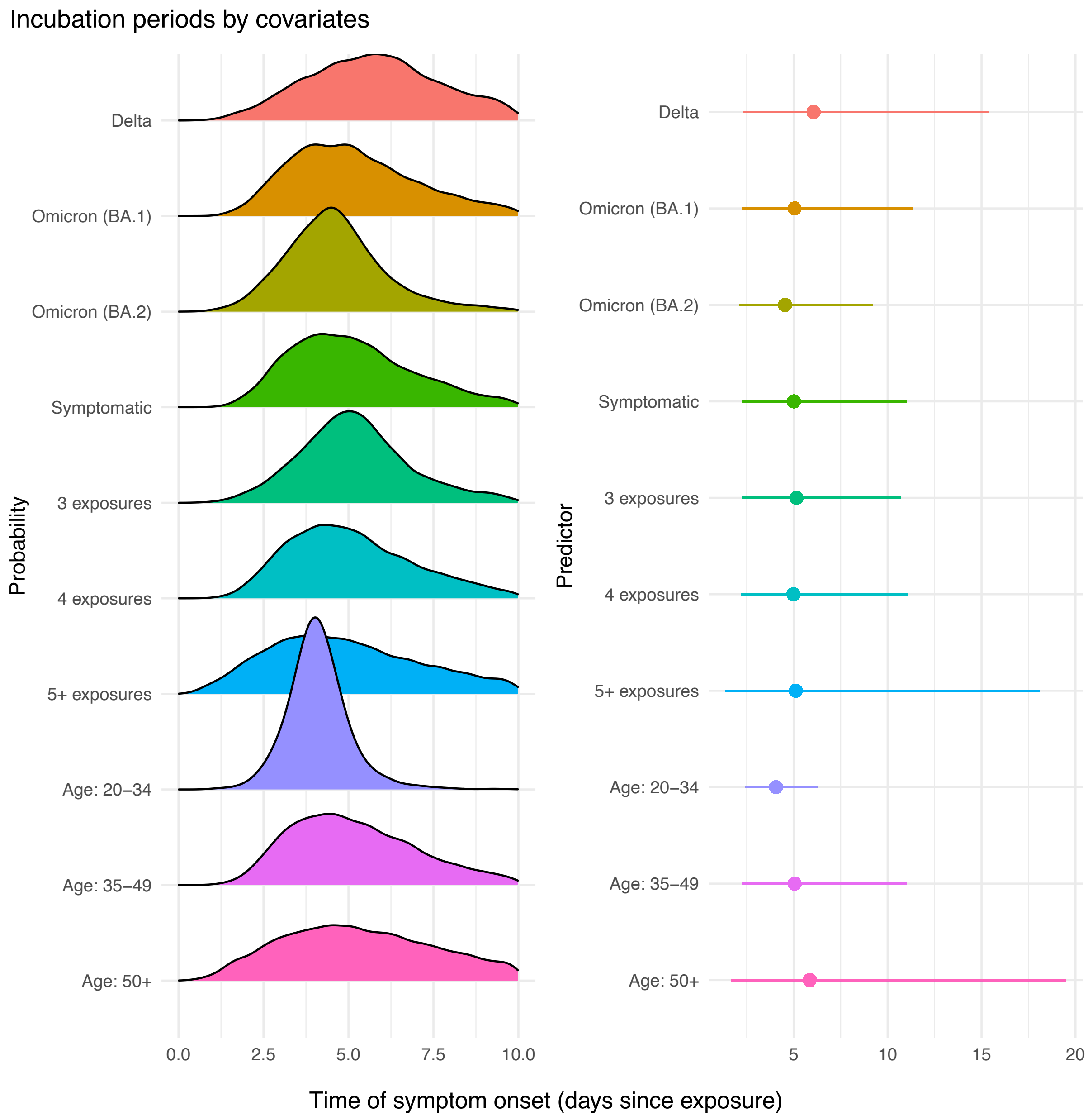

Fig V. Density and effect size plots of the inferred incubation periods, for each covariate. A: Incubation periods generated using the inferred posterior distributions for both the mean and standard deviation, stratified by each covariate. **B.** The mean (dots) and 95% CrI of the incubation periods plotted in **Panel A.**

Gene target and swab type adjustment

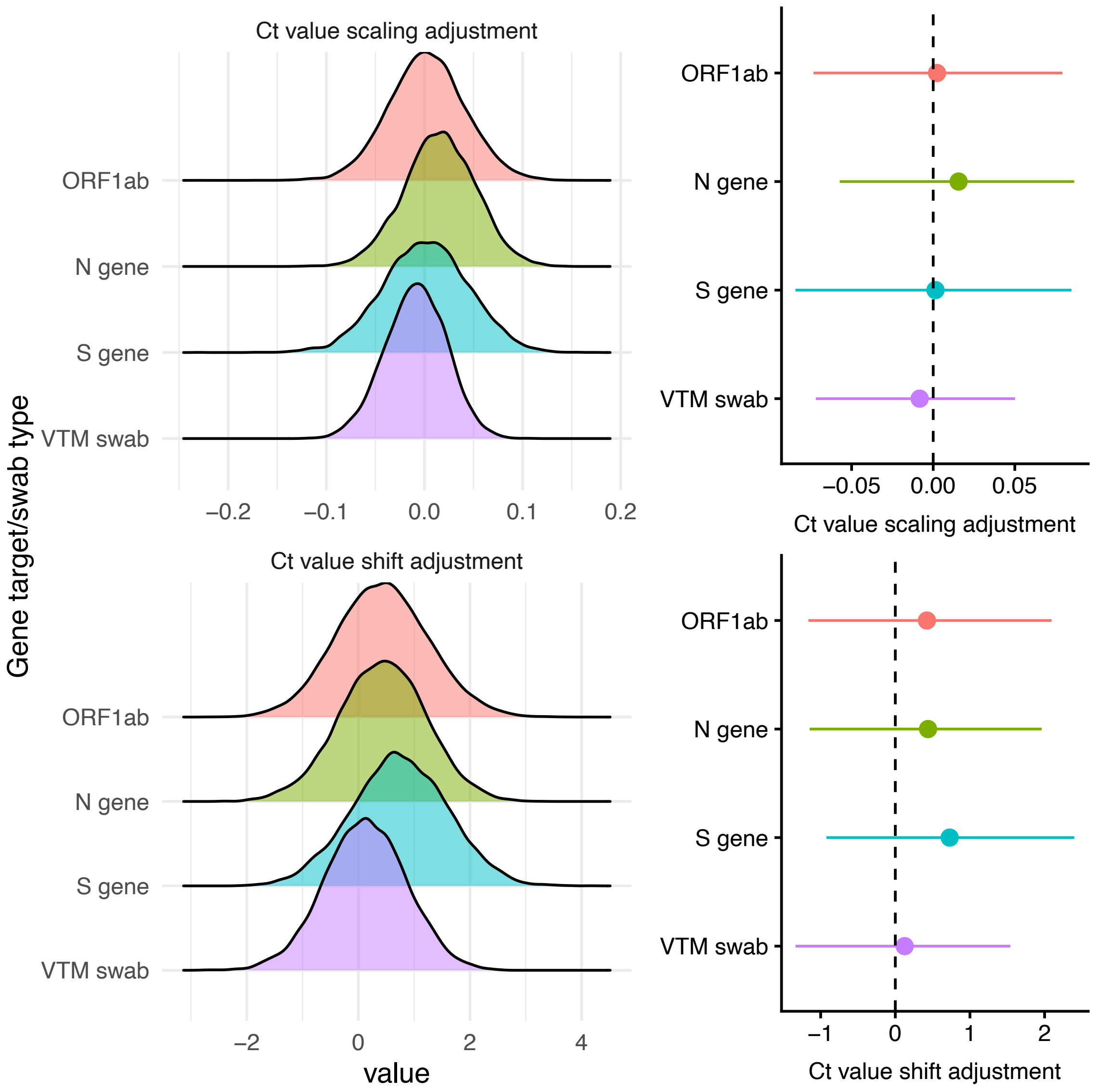

Fig W. Posterior density plots for the estimated effect sizes for the Ct adjustment component of the model. We plot the posterior distributions (left panel) and the median and 95% credible interval (right panel) for the adjustment factors for the Ct scale and Ct shift parameters, stratified by each Ct target and swab type.

Population-level prior vs posterior comparison

To directly investigate the influence of our choice of priors and our model (conditioned on our dataset) used in the main results, we plot the population-level priors (equivalent for each predictor) against the inferred posteriors, by the predictors used in the main results. The inferred posterior distributions clearly differ from the priors in location and width for all parameters and covariates, showing that the posterior distributions are strongly informed by the data.

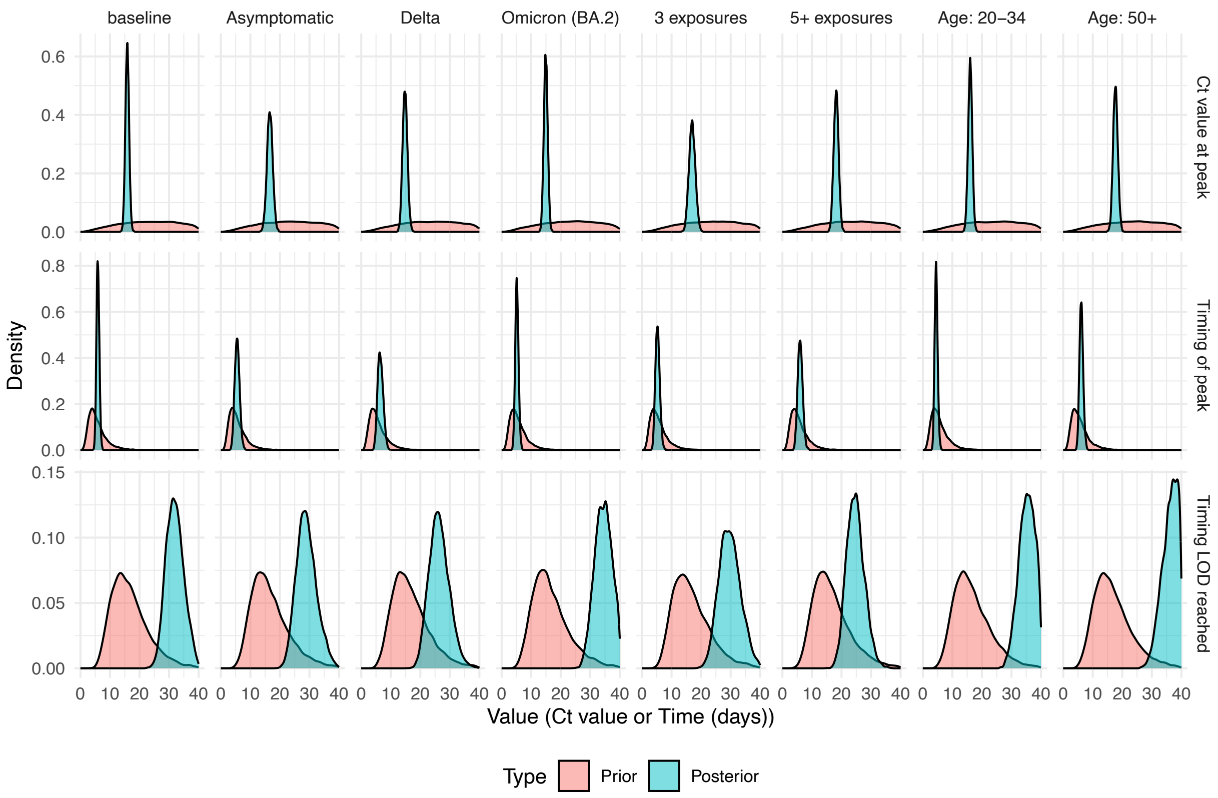

Fig X. Population-level priors and inferred population-level posterior distributions for peak Ct value (cp), time to peak Ct value (tp), and time to LOD (tlod) by each covariate used in the main results.

Simulation study

To test the ability of our modelling framework to recover known parameter values, we simulated two groups of individual-level data, whereby one group was treated as the baseline case and the other included in our regression framework. Specifically, the simulation process performed was as follows:

1. Draw population-level parameters from the priors for the key process parameters in question: the Ct value at the peak, the timing of the peak and the time that the LOD is reached.
2. Multiply the parameter values by three factors, which we assume are the ground-truth multiplicative differences between the process parameters for the baseline group and two other synthetic groups of individuals.
3. Generate individual-level parameters using the population-level parameter draws and draws from the priors of the individual-level variation and covariance structure.
4. Simulate individual-level trajectories for N = 10 and N = 20 individuals using the individual-level parameters.
5. Sample from their trajectories with a mean sample density equivalent to that in the true dataset (mean negative tests = 1.5 and mean positive tests equal to 6).
6. We fit the model to the simulated data, including the two groups as a covariate in the inference.

We found that for N = 10 individuals, the direction of the effect size is consistently recovered, with well-informed posteriors relative to the priors (**Fig Y**). The quality of the recovery is mixed. The posterior is typically centred around the true value, but with high uncertainty, given the low statistical power. However, for N = 20, we find that the direction and magnitude of the true effect size is consistently recovered (**Fig Z**). Given that out of the many groups considered in our main analysis the number of individuals in the smallest group in our dataset is the asymptomatic group in the Symptom status category (N = 12), with the next smallest group bigger than 20 (N = 26), we believe that our study population and modelling framework does allow us to disentangle the various confounding factors.

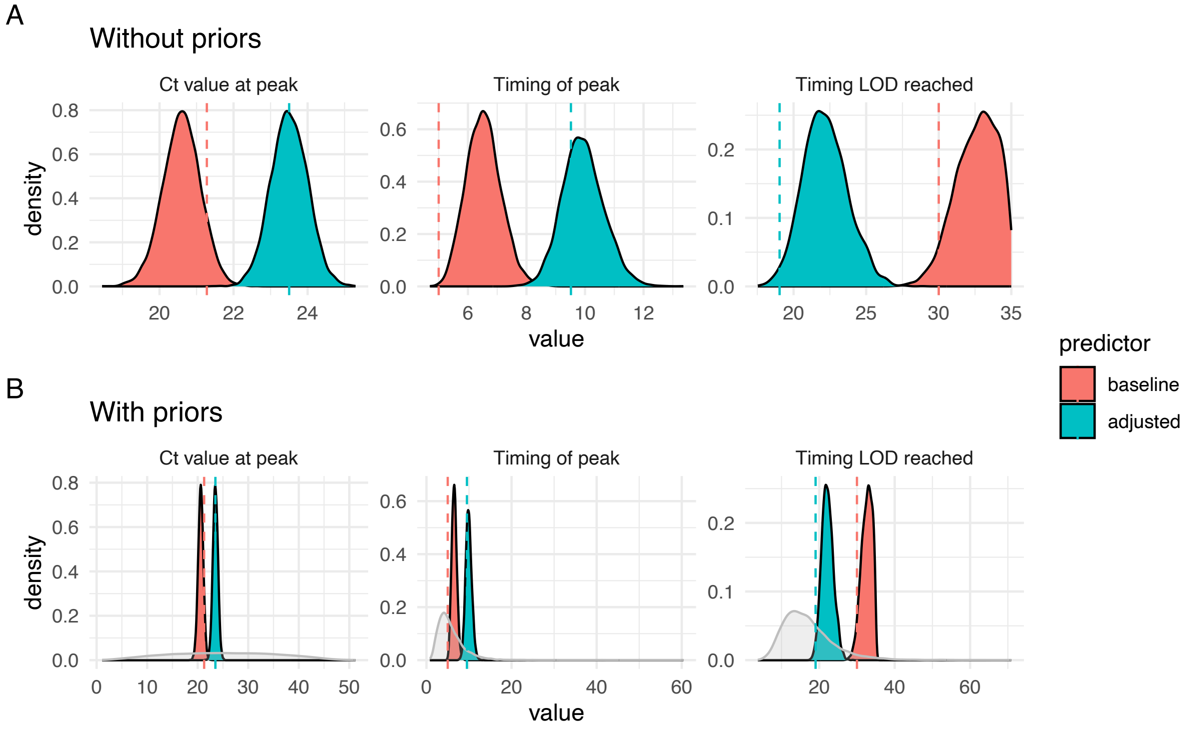

Fig Y. Parameter recovery after fitting to a small, simulated dataset. We fit the model to an artificial dataset for N = 10 individuals, using parameters sampled from the semi-informative priors used in the main analysis. We see reasonable recovery of the true effect size parameters, recovering the direction of the effect well, but not fully able to recover the true magnitude, given that the dataset is small.

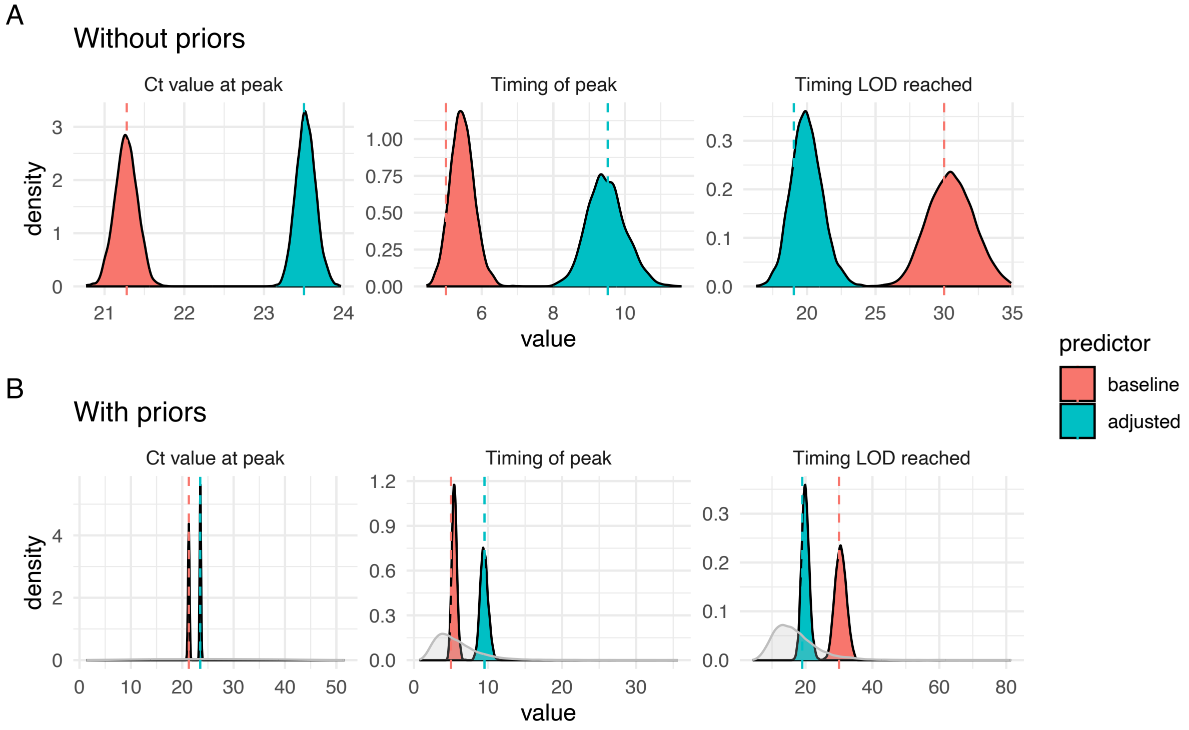

Fig Z. Parameter recovery after fitting to simulated data. We fit the model to an artificial dataset for N = 20 individuals, using parameters sampled from the semi-informative priors used in the main analysis. We see good recovery of the true effect size parameters.

Prior predictive distribution

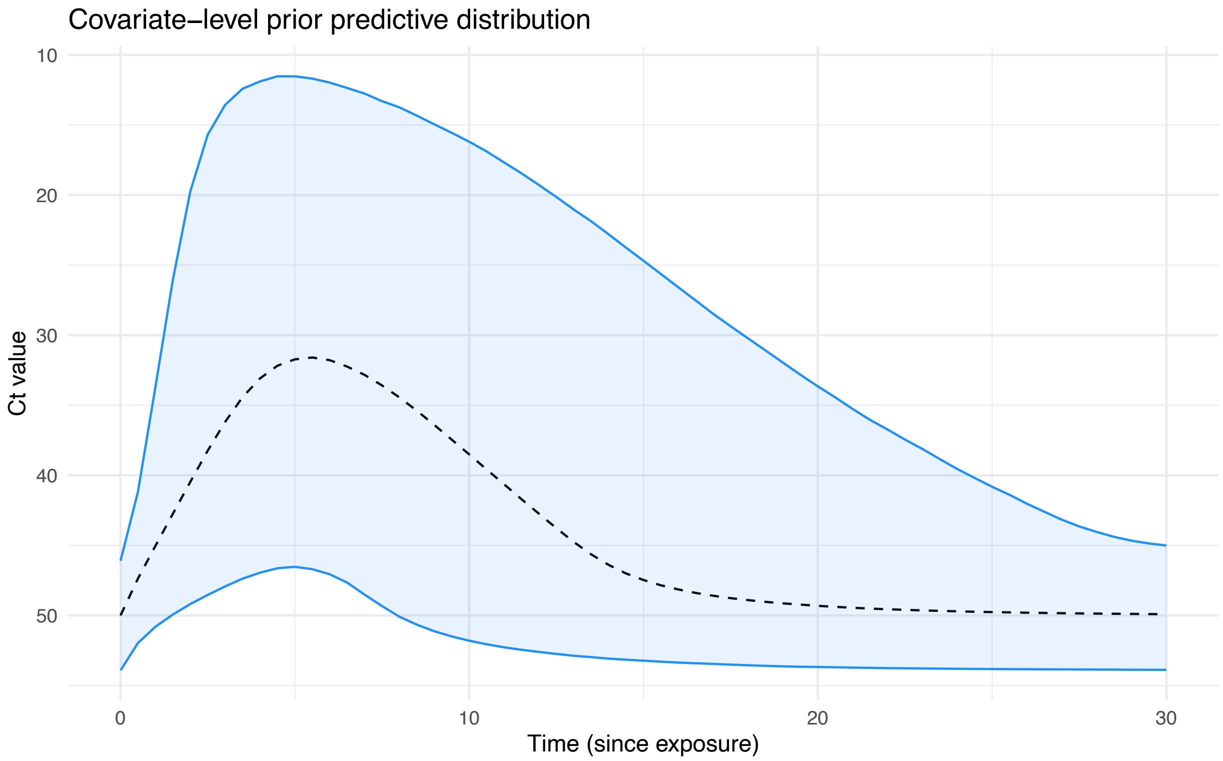

Fig AA. Prior predictive distribution. We calculate the prior predictive distribution produced using the covariate-level prior distributions. We do so by drawing 100,000 samples from the covariate-level priors, generating the corresponding viral kinetic trajectories, and summarising them by calculating the median and 95% credible intervals. We do so to clearly show the range of trajectories our semi-informative priors produce, without the extra individual-level variation and multivariate normal structure.

Leave-one-out model comparison

To justify the inclusion of the covariates used in the main analysis (VOC, symptom status, number of exposures and age group), we performed a leave-one-out (LOO) analysis using the loo package in R (8). The loo package approximates a full LOO analysis (where the model would need to be refit N – 1 times, where N is the total number of Ct values in this case, which would be computationally expensive. The package uses Pareto-smoothed importance sampling, which requires the model to be fit just once for each model type. It gives an expected log-predictive density (ELPD) value for each model fit and a standard error (SE) value associated. Once all model candidates are fit, it gives the ELPD-diff and SE- diff to allow straightforward comparisons between model structures. **S7 Table** shows the ELPD and associated SEs for all the model candidates considered.

Primarily, we were interested in assessing the inclusion of the VOC covariate, given that there is a strong a priori biological justification for VOC having some effect on shedding, but we want to avoid the risk of overfitting that comes with a more complex model. We found that some models with a single covariate fit at a time perform worse than the model with all covariates and some better, although the differences and standard errors are small between all model candidates. A lower bound for a reliable difference between two model structures is a 4-fold difference between the LOO-IC (which is -2 x ELPD) and the standard error difference, which is not the case between any of our model candidates. Therefore, we conclude that there is no penalty incurred in terms of LOO predictive performance for the more complex VOC model, and potentially even a small advantage. The table reporting the results from the full LOO analysis can be found at **S7 Table**.

S7 Table. Full table of ELDP, ELDP differences, standard errors, and SE differences, as computed using the loo function incorporated in the cmdstanr R package. The models are reported in no particular order.

References

1. Hay JA, Kissler SM, Fauver JR, Mack C, Tai CG, Samant RM, et al. Viral dynamics and duration of PCR positivity of the SARS-CoV-2 Omicron variant [Internet]. Epidemiology; 2022 Jan [cited 2022 Jun 20]. Available from: http://medrxiv.org/lookup/doi/10.1101/2022.01.13.22269257

2. Kissler SM, Fauver JR, Mack C, Tai CG, Breban MI, Watkins AE, et al. Viral Dynamics of SARS-CoV-2 Variants in Vaccinated and Unvaccinated Persons. N Engl J Med. 2021 Dec 23;385(26):2489–91.

3. Hay JA, Kissler SM, Fauver JR, Mack C, Tai CG, Samant RM, et al. The impact of immune history and variant on SARS-CoV-2 viral kinetics and infection rebound [Internet]. medRxiv; 2022 [cited 2022 Aug 18]. p. 2022.01.13.22269257. Available from: https://www.medrxiv.org/content/10.1101/2022.01.13.22269257v2

4. Lauer SA, Grantz KH, Bi Q, Jones FK, Zheng Q, Meredith HR, et al. The Incubation Period of Coronavirus Disease 2019 (COVID-19) From Publicly Reported Confirmed Cases: Estimation and Application. Ann Intern Med. 2020 May 5;172(9):577–82.

5. Killingley B, Mann AJ, Kalinova M, Boyers A, Goonawardane N, Zhou J, et al. Safety, tolerability and viral kinetics during SARS-CoV-2 human challenge in young adults. Nat Med. 2022 May;28(5):1031–41.

6. R Core Team. R: A Language and Environment for Statistical Computing [Internet]. Vienna, Austria: R Foundation for Statistical Computing; 2021. Available from: https://www.R-project.org/

7. stan-dev.github.io [Internet]. [cited 2023 Mar 24]. Documentation. Available from: //mc-stan.org/users/documentation/

8. loo.pdf [Internet]. [cited 2023 Oct 2]. Available from: https://cran.r-hub.io/web/packages/loo/loo.pdf
