# Supplementary material for: Combined analyses of within-host SARS-CoV-2 viral kinetics and information on past exposures to the virus in a human cohort identifies intrinsic differences of Omicron and Delta variants
Source: PLoS Biol. 2024 Jan 30;22(1):e3002463. doi: 10.1371/journal.pbio.3002463 (PMC10826969; doi:10.1371/journal.pbio.3002463)
Supplement: Attachment — Submitted filename: Reply to reviewers comments.docx [file pbio.3002463.s009.docx]

Reply to reviewers' comments for:

## Within-host SARS-CoV-2 viral kinetics informed by complex life course exposures reveals different intrinsic properties of Omicron and Delta variants

Dear Dr. Russell,

Thank you for your patience while your manuscript "Within-host SARS-CoV-2 viral kinetics informed by complex life course exposures reveals different intrinsic properties of Omicron and Delta variants" was peer-reviewed at PLOS Biology. It has now been evaluated by the PLOS Biology editors, an Academic Editor with relevant expertise, and by several independent reviewers.

In light of the reviews, which you will find at the end of this email, we would like to invite you to revise the work to thoroughly address the reviewers' reports.

Reply:

We thank the editors and the reviewers for their evaluation of our manuscript. We have revised the paper in line with all of the suggestions from the editor and the reviewers.

As you will see below, the reviewers think that the manuscript is interesting but they find some issues related with your data analyses and interpretation of the results that should be solved before publication. We think that a clearer exposition of the statistical analysis would benefit the paper. In particular, we agree with the reviewers that you should double check for the significance of the variant effects by testing your models against models without variants as a cofactor, and also to discuss the estimated effects sizes in comparison with previous estimates. This is particularly relevant as the difference between Delta and Omicron variants are highlighted in the title, while the abstract highlights mostly effects of age and prior exposure.

Reply:

We realise that the exposition of the statistical analysis could have been clearer. We have reworked the model description section, separating out the underlying model structure and the assumed priors used. We have also performed a number of additional robustness checks, including more sensitivity analyses, a simulation study showing that our inference framework can recover effect sizes with sample sizes similar to the sizes in our smallest subgroups (and smaller) and a full model selection procedure, in line with the editors’ and reviewers’ comments. We find the model with VOC as a covariate has higher predictive power relative to the other model structures considered — without VOC and with each covariate at a time — providing justification for its inclusion in the study, given its high biological significance.

We also agree it would be useful to have some more context on the differences in overall effect size between this study and previous studies. We now include biological, experimental, and methodological discussion around why such differences may occur.

As a side note, we would like to acknowledge that we have been able to include 17 more infection events in our analysis. This is due to a misclassification of symptom statuses within the dataset. Infection events were being filtered out due to missing symptom status attributions. However, individuals had symptom onset dates. After long discussions with our collaborators, they were able to reconcile the differences. As such, we included them in our analysis. None of the conclusions of our study have changed. However, many of the numerical values have been updated. The main effect it has had is tightening the credible intervals we were able to estimate. This is the reason for many of the tracked changes within the results section where we quote specific results.

Given the extent of revision needed, we cannot make a decision about publication until we have seen the revised manuscript and your response to the reviewers' comments. Your revised manuscript is likely to be sent for further evaluation by all or a subset of the reviewers.

We expect to receive your revised manuscript within 3 months. Please email us (plosbiology@plos.org) if you have any questions or concerns, or would like to request an extension.

At this stage, your manuscript remains formally under active consideration at our journal; please notify us by email if you do not intend to submit a revision so that we may withdraw it.

**IMPORTANT - SUBMITTING YOUR REVISION**

Your revisions should address the specific points made by each reviewer. Please submit the following files along with your revised manuscript:

1. A 'Response to Reviewers' file - this should detail your responses to the editorial requests, present a point-by-point response to all of the reviewers' comments, and indicate the changes made to the manuscript. *NOTE: In your point-by-point response to the reviewers, please provide the full context of each review. Do not selectively quote paragraphs or sentences to reply to. The entire set of reviewer comments should be present in full and each specific point should be responded to individually, point by point.

You should also cite any additional relevant literature that has been published since the original submission and mention any additional citations in your response.

2. In addition to a clean copy of the manuscript, please also upload a 'track-changes' version of your manuscript that specifies the edits made. This should be uploaded as a "Revised Article with Changes Highlighted" file type.

*Re-submission Checklist*

When you are ready to resubmit your revised manuscript, please refer to this re-submission checklist: https://plos.io/Biology_Checklist

To submit a revised version of your manuscript, please go to https://www.editorialmanager.com/pbiology/ and log in as an Author. Click the link labelled 'Submissions Needing Revision' where you will find your submission record.

### Reviewers' comments

**Reviewer #1**

**Summary**

The study by Russell et al. proposes an analysis of viral kinetics in individuals infected by Omicron or Delta variants. Despite the millions of people that have been infected by these viruses, the specific patterns of viral kinetics associated with these infections remain obscure. This is due to the fact that analysing retrospective cohorts of infected individuals, typically sampled after symptom onset and when viral load is already in its clearance phase, is prone to many biases and confounding factors: immunity levels, vaccination rate, incubation period, testing policy…therefore, the only study design to properly address this question is to constitute a cohort of uninfected individuals followed longitudinally, frequently tested to detect early infection, and in whom positive PCR triggers and even more frequent sampling of viral load. This is exactly what the authors have designed, at least in a subset of their population, and this is what makes this study novel and interesting. Such design is however expensive and difficult to implement, relying on a less captive population of outpatients, and the data presented, albeit being very original, remain limited to address the complex question of viral kinetics. Overall the effect size that are found seem very limited, suggesting no major differences across variants. As such some of the results raise concerns about the possibility to really extrapolate from this population, and I am not totally convinced that the studied population is sufficiently large to really disentangle the different

confounding factors.

Reply:

We are glad that the reviewer also appreciates the novelty and value of our analysis. We agree that this kind of study design is the only way to properly address this question, but it can still be challenging to fully understand how factors combine to influence shedding. We elaborate more in responses to the reviewers’ comments below, but to investigate the point about population size, we have now included the results of a simulation study in the revised supplementary material (**Figure S25, S26**). This allowed us to test the ability of our approach to recover the true simulated effect for a given number of individuals within each group. The overall effect size and its associated uncertainty in a fully Bayesian analysis, such as the one performed in this study, is influenced by a number of sources of uncertainty, including: the uncertainty from the semi-informative priors, the overall volume of data and the size of the true underlying effect sizes relative to the priors, and the model specification. In our simulation study we found that when fitting the model to simulated data with known large effect sizes but small overall numbers of individuals and data points (smaller than in each group used in our full analysis), we obtained posteriors that are still well-informed and recover the known effect sizes. We therefore believe the effect sizes estimated in our analysis are unlikely to be purely an artefact of limited data (e.g. apart from perhaps 1-2 subgroups as shown in Figure S4) and that they reflect the magnitude of effect sizes present within the data, after adjusting for the interactions between the various covariates.

The revised supplementary material describes the simulation study performed in a new section, reading:

*To test the ability of our modelling framework to recover known parameter values, we simulated two groups of individual-level data, whereby one group was treated as the baseline case and the other included in our regression framework. Specifically, the simulation process performed was as follows:*

1. *Draw population-level parameters from the priors for the key process parameters in question: the Ct value at the peak, the timing of the peak and the time that the LOD is reached.*
2. *Multiply the parameter values by three factors, which we assume are the ground-truth multiplicative differences between the process parameters for the baseline group and two other synthetic groups of individuals.*
3. *Generate individual-level parameters using the population-level parameter draws and draws from the priors of the individual-level variation and covariance structure.*
4. *Simulate individual-level trajectories for N = 10 and N = 20 individuals using the individual-level parameters.*
5. *Sample from their trajectories with a mean sample density equivalent to that in the true dataset (mean negative tests = 1.5 and mean positive tests equal to 6).*
6. *We fit the model to the simulated data, including the two groups as a covariate in the inference*

*We found that for N = 10 individuals, the direction of the effect size is consistently recovered, with well-informed posteriors relative to the priors (****Figure S25****). The quality of the recovery is mixed. The posterior is typically centred around the true value, but with high uncertainty, given the low statistical power. However, for N = 20, we find that the direction and magnitude of the true effect size is consistently recovered (****Figure S26****). Given that out of the many groups considered in our main analysis the number of individuals in the smallest group in our dataset is the asymptomatic group in the Symptom status category (N = 12), with the next smallest group bigger than 20 (N = 26), we believe that our study population and modelling framework does allow us to disentangle the various confounding factors.*

The revised manuscript refers to this new analysis in the results section:

*To test the sensitivity of our model to the choice of semi-informative priors, the sample sizes, and our choice of covariates, we performed a number of additional robustness checks, sensitivity analyses and a model selection procedure…*

*…To test the ability of our model to recover known parameter values, we performed a simulation study (described in detail in the Supplementary Material). Figures S25 and S26 show results after fitting our model to simulated data whereby we demonstrate the ability of our modelling framework to recover known effect sizes varying three key process parameters between two synthetic groups smaller than or similar in size to the smallest group of individuals in our analysis.*

**Comments**

It is nice to have conceptual figures but figure 2 is probably too complex. The originality of the study lies in the population of individuals that joined before infection episode (N=75) and I would suggest to add simple information focusing on this population as follows:

Summary statistics: number of points available per patient, delay between first viral load data and symptom onset, observed peak viral load, time to clearance and basic statistical comparisons between the 3 viral strains. Even if there are a number of confounders (that will be addressed by modelling) please show viral load data available at different time points for each variant and basic summary statistics to compare variants. If data are sparse, you can for instance bin timepoints (every two of three days). The time reference could be the time since first positive PCR. This would help appreciate the quantity of info available (and probably would be more informative than the colour code used in figure 2).

We agree that there is a balance to be struck between detail and accessibility in Figure 2 and considered several versions while developing the original manuscript. Based on the reviewers’ comments, we have tried to streamline the presentation of the data by further refining the figure.

As our dataset includes a range of covariates that we use in our inference framework, the primary aim of Figure 2 was to display the data we use in the main analysis. In particular, we wanted to illustrate that our inference framework fits to individual-level data and pools the estimates in a hierarchical framework to arrive at estimates stratified by covariates (Figures 3 and 4). However, some of these individual-level data may be better placed in the supplement. Therefore, we have moved panel B of Figure 2 to the supplementary material (Figure S2) and replaced it with a figure of the same data stratified instead by VOC, not by individual. We believe the new version of these figures gives a more reader-friendly representation of the same data.

In terms of time points, the second panel of this figure is already plotted by time-since-first-positive-test on the x-axis (panel B). However, we agree it would be useful to provide some summary statistics about the subset of the population who were naive when entering the study. We have added a table to the supplementary material with the statistics requested (Table S1) for this subpopulation. Furthermore, we plot the same quantities as we show in the table for this subpopulation in four new simple figures (Figures S3) and the same quantities for the entire cohort (Figure S4). We have added the following paragraph to the supplementary material:

*Over half of the individuals within the study had not reported an infection before they entered the study. For those individuals categorised as infection naïve, viral load dynamics may differ to previously infected individuals. We account for this in the model in two ways: we consider the total number of exposures (the sum of infections and vaccines for each individual), as well as the time since the most recent exposure for each individual as covariates in our model. Because prior infections can be included in the analysis via the exposure covariate, we did not focus solely on infection naïve individuals. However, we include a table of the summary statistics for the infection naïve individuals (****Table S1****), along with a new figure each with four panels plotting the following: the number of points available per patient, the delay between first viral load data and symptom onset, the observed peak viral load, and the time to clearance, all stratified by the VOCs considered in this study (****Figure S3****). Lastly, Figure S4 has the same structure and layout as Figure S3 but showing data from the entire cohort.*

I am fine with Bayesian analyses but again very difficult to appreciate to what extent results are sensitive to prior distributions. Please provide the prior distributions that were used, and in case they are "semi-informative".

We originally showed the covariate-level priors alongside the posteriors in the supplementary material (originally Figure S16). That figure showed substantial deviation away from the priors once they had been conditioned on our data (under the assumptions of our model). However, we now include three new figures (Figures S13—S15) which show the individual-level priors and posteriors on the same plots (one plot for each VOC). We include a new paragraph in the revised version of the supplementary material which reads:

*In a similar style to the individual-level posterior distribution plots (****Figures S6—S8****), we plot the individual-level prior distributions on the same plot as the posterior distributions, to indicate the overall effect our semi-informative priors have on our overall posterior distributions. As can be seen from the new figures, the posterior distributions are well-informed relative to the semi-informative priors and where the data influences them, away from the values with high density within the priors.*

We do so to show how far the posteriors have moved away from the priors. Furthermore, we now show a covariate-level prior predictive distribution in a new figure (Figure S20), which outlines the range of possible trajectories for the viral kinetics model. We include a new subsection in the revised supplementary material describing this new plot, which reads:

*We calculate the prior predictive distribution produced using the covariate-level prior distributions. We do so by drawing 100,000 samples from the covariate-level priors, generating the corresponding viral kinetic trajectories, and summarising them by calculating the median and 95% credible intervals (****Figure S27****). We do so to clearly show the range of trajectories our semi-informative priors produce, without the extra individual-level variation and multivariate normal structure.*

I would recommend conducting a sensitivity analysis to evaluate to which extent results were dependent on the prior, and what would be the results in case of uninformative priors. This would help modellers appreciate what can be reliably inferred from these data, and what requires to borrow from literature.

We have added a new figure (Figure S20) which shows the model when fit with uninformative priors. We have added the following text to the revised supplement describing the new results:

*To investigate how sensitive our results from our main analysis are to our semi-informative priors, we widen the covariate-level priors by increasing the variance parameter considerably for the three main process parameters: Ct value at peak, timing of the peak and timing the LOD is reached. We still used Normal distributions for all covariate-level priors, as they are highly recommended for high-dimensional hierarchical models such as this one, to aid with sampling from complex geometries. However, where the variance parameter was between 0.5 and 1 for all semi-informative priors — with mean values chosen from literature — we multiply all variance parameters by 5, widening the priors considerably. The parameters are on logit (Ct value parameters) or log scales (timing parameters), meaning that this increase in the variance when measured in natural units (Ct values or time (days)), should also be interpreted on these same scales. The results using uninformative priors are in line with the semi-informative priors (****Figure S20****). The direction of all effect sizes is the same between the two and all magnitudes are within each other’s credible intervals. The overall uncertainty for the uninformative prior case is slightly wider, but at a level hard to distinguish visually. We conclude from this that our results from our main analysis using semi-informative priors are not overly sensitive to the choices made and that our resulting estimates are mostly informed by our modelling framework conditioned on our dataset.*

Also, using the leave-one-out model comparison also added to the revised study, we show that the model with uninformative priors has the lowest predictive power of all model candidates, indicating that model performance benefits from a combination of biologically informative priors alongside inferential power from the data itself. We use the combined fact that the central model estimates from the model with non-informative priors are similar to the model with semi-informative priors, just with substantially wider uncertainty, to justify the inclusion of semi-informative priors in the main analysis. We have added the following paragraph to the discussion of the revised manuscript to describe the extra analysis.

*To test the relative predictive performance of several model candidates, we performed a leave-one-out (LOO) model selection procedure for eight candidate models (see Table S7 for the candidate models considered). The candidate models have either different design matrices, specifying which set of covariates are included in each model, or different priors to the model used in the main analysis. The predictive power of all models included in the LOO analysis with different design matrices are within four standard errors of one another, which is broadly the rule of thumb for a model with significantly lower predictive power than another. Therefore, we can conclude that including covariates known to influence viral kinetics is justified, given that the models have similar predictive power with or without their inclusion. The model with non-informative priors, which has the lowest predictive power compared to all other candidates —relative to the model with semi-informative priors used in the main analysis (****Table S7****) — is still within the range where it is difficult to conclude an overall difference in predictive power. As such, we also performed a complementary sensitivity analysis in which we plot the effect sizes of the model with non-informative priors (****Figure S20****). The central effect size estimates are broadly similar in both the semi- and non-informative models (****Figure 3, Figure S20****). However, the resulting uncertainty is higher in the non-informative prior case. Combining the interpretation from the LOO and sensitivity analysis around the influence of semi-informative priors, we conclude that their inclusion in the results reported in the main analysis is justified, given that the predictive power is higher with them, the central estimates are broadly similar and prior information is only used where reliably reported elsewhere in published literature.*

Some of the results seem in contradiction with the literature which raise concern about the extrapolation that can be made from the data. For example, there is a large body of data suggesting that age is associated with extended time to viral clearance. I find it very surprising that age groups 20-34 and 50+ seem to have similar time to viral clearance. It is possible that this is due to the fact that the model accounts for repeated exposure, which is not done usually but still seems very counterintuitive. See for instance model based approaches in Hay et al, Elife (2022); Neant et al., PNAS (2021).

Same thing for the peak viral load. It was my understanding that delta was associated with higher peak viral load than BA.1. Perhaps not in the order published initially (*1000) but not as small as suggested here (1 Ct, eg, 2 fold). For instance in Hay et al, the difference seems to be ~1 log (Table S5). In the macaque model (https://www.biorxiv.org/content/10.1101/2022.11.09.515748v1), the difference was also larger (close to 2 logs, Table S1). I understand the difficulty to arrive at a consensus value given all the confounders, but I think that the authors should acknowledge that some of these results are not necessarily consensual, and should discuss what could explain the discrepancy between their results and those of the literature.

We agree it would help to expand the discussion of our results in the context of other findings, which were often performed with different study designs and in different populations. We have amended an existing paragraph and added two new paragraphs to the discussion of the revised manuscript to highlight these differences and possible explanations. The revised section of the discussion, starting in the revised manuscript, now reads:

*Previous studies inferring viral kinetics have concentrated mostly on differences between VOCs circulating at the time in question. As such, they did not consider the host of other possible confounding variables, making their results difficult to attribute solely to differences in infecting VOC (7, 13, 22, 40). In interpreting effect sizes between viral kinetic estimates, we controlled for the complex emerging picture around individual life-course exposures to SARS-CoV-2, both as infections and vaccines. Differences in numbers of infections, the VOC causing the infection, the numbers of vaccinations, and many other individual-level variables introduce many sources of possible confounding. In particular, the timing of the sampling within our study differs compared to other studies, and hence Ct dynamics by variant could vary substantially across populations if these factors are not adjusted for.*

*Moreover, we find less difference in viral kinetics by age in comparison to other studies [references]. Again, this is likely to be influenced by a number of factors. Given the complex relationship between age, symptom severity, immunity and exposure risk, studies that do not account for these characteristics could attribute differences in shedding to age, rather than another factor that correlates with age. However, combining the underlying viral kinetics model with a Bayesian hierarchical framework allowed such information to be jointly incorporated into our analysis. Additional factors that could influence observed differences in effect size estimates include: calibration differences between PCR pipelines resulting in differences in the absolute Ct values; differences in sampling procedure with previous studies primarily relying on symptom-triggered sampling; complex exposure histories following both the vaccine rollout and the emergence of new VOCs and ability of the underlying model structure to account for the numerous covariates suspected to affect viral kinetics or the inherent individual-level heterogeneity in response to exposure to SARS-CoV-2. We combine data from a study designed to capture viral load across entire infections, including sampling before and after exposure with a model structure complex enough to adjust for all necessary covariates, individual-level variation in viral kinetics and able to inform model parameters by jointly fitting to data where possible. Therefore, the differences between our effect sizes and previously published studies may be explained by the combined adjustment for all of these complexities.*

*The flexibility provided by our framework provides a methodological foundation for future work using similar datasets. Given the ongoing antigenic turnover of SARS-CoV-2 variants of concern since late 2020, our results show it will be increasingly important to adjust for a range of changing host factors when quantifying viral kinetics and considering implications for potential infectiousness in future.*

Going back to my first point, perhaps it would also be nice to discuss the differences between the model-based approach and the crude summary statistics of the data. It is possible that the authors show that it is precisely the model based approach that allows adjusting some metrics and could explain some of the discrepancy with more descriptive approaches.

We agree that the benefit of a full model structure for such a rich dataset could be more clearly outlined in our study. We have remade Figure 2 to more clearly show the difference between Ct values of the raw data in comparison to our estimated Ct values (Figure 3). We believe this allows the reader to see the benefit of fitting latent trajectories to data with highly differing sampling densities and high levels of individual-level variation.

**Reviewer #2**

**Summary**

In this manuscript, the authors have fitted a mathematical model of viral kinetics for SARS-CoV-2 kinetics, which they have fitted to data from an observational cohort study of health adult volunteers. This is a very interesting piece of work: the dataset is detailed and complex, and the modelling has been developed and carried out in a way that reflects this. In general, the methods and results are well presented. I have a few concerns and questions, especially around interpretations of the results, which should be addressed. Nevertheless, I commend the authors on an interesting study.

We thank the reviewer for their summary of our study, especially for recognising that the work contains both a complex and rich dataset and a modelling approach which reflects it.

**Comments**

Personally, I wouldn't describe the model as mechanistic. The viral load model is a piece-wise regression model: no mechanism is proposed for why the exponential growth of the virus (linear decrease of the Ct values) stops (resource depletion, immune response, etc.), and viral load then declines. However, these terms are used differently in different fields: I'll leave the final choice of this to the authors.

Although the model does not capture the full underlying within-host dynamics, a piecewise linear model includes more mechanistic knowledge than straight forward regression. Exponential growth and decline on the natural scale as predicted by within-host models is equivalent to a piecewise linear model on the log scale. We therefore reserve the term ‘regression’ for the part of the model which is entirely statistical: the effect sizes, describing differences in process parameters at the covariate level. However, we realise it may be clearer to avoid any doubt and change the *mechanistic* model to the *viral kinetic* model in both the revised manuscript and revised supplementary material.

I'm a little unclear on the definition of the incubation period: in Figure 1B, it appears to be the time between exposure and the peak viral load. However, lines 182-186 in the manuscript suggest it is the time difference between exposure and symptom onset. Can this be clarified?

We thank the reviewer for pointing out this ambiguity. The definition of the incubation period used throughout the analysis is the same as the standard definition: the difference in time between exposure to the virus and the onset of symptoms. However, in the original version of Figure 1, it looks as though we are defining it to mean the difference between the exposure time and the timing of the peak of the viral load. We have remade panel B of Figure 1 in the revised manuscript so that it depicts the correct definition of the incubation period.

I'm a bit confused about the various censoring used in the model likelihood. There is a truncation below Ct = 0: presumably a PCR machine is incapable of recording a negative number of cycle thresholds? I would say that the likelihood should instead be truncated at zero, but I'm not sure this is an important distinction. Based upon figures like Fig S6, it looks like very few (if any) datapoints show Ct < 10, so I don't think this is influencing the model results. The censoring at Ct > 40 is a more important, however, and has confused me. It is unclear to me if this represents the limit of detection (LOD) of the PCR machine(s) used in this study, or whether it is simply the smallest Ct value recorded in the dataset? The latter might explain why the authors are also trying to infer an additional LOD parameter. Additional clarification is needed here.

We thank the reviewer for their deep dive into the model structure used in our inference framework. We make the distinction between truncation and censoring explicit in our updated supplementary material. As such, the threshold Ct = 0 is a truncation threshold, given that PCR machines can not report values less than 0. Given that we use only semi-informative priors, some trajectories towards the initial samples of the inference do produce expected Ct values that can go below 0, which justifies the inclusion of this term. However, given that the data informs the inference away from such parameter estimates, the term only contributes a small amount to the overall likelihood. The censoring threshold of Ct >= 40 is more important. It does indeed correspond to the LOD of the PCR machines used in this study. We infer an additional LOD parameter which corresponds to the latent (typically very high, with a mean value of Ct = 50 specified in the semi-informative priors) Ct value which would happen between the times of exposure and time the virus is detectable by a realistic machine. This allows us to estimate the latent Ct trajectory at all time points, in particular between exposure and detectability. The inclusion of this parameter is, we believe, an important extension made in this study compared to previous studies, as it allows the full Ct trajectory to be estimated — not just at the times after the trajectory has passed the LOD of the realistic PCR machine. We have added the following sentence to the revised supplementary material to clarify all of these points:

*The process parameters c_e_ and c_lod_ represent the Ct value at the moment of exposure. This is a latent quantity as the virus would not be practically detectable by any realistic PCR machine at this time. However, it helps us frame and interpret the model by including it for each individual, as it allows us to explicitly model the timing of exposure for each individual. The process parameters c_e_ and c_lod_ represent the Ct value at the moment of exposure. This is a latent quantity to be estimated, as it is unrealistic to expect it to be observable, given that the virus would not be practically detectable by any realistic PCR machine at this time. However, it helps us frame and interpret the model by including it, as it allows us to explicitly model the timing of exposure for each individual. These parameters differ from the LOD of the PCR machine used, which was Ct = 40.*

I found the description of the hierarchical model hard to follow. Line 127 of the S.I. presents the Ct kinetics model, with 6 parameters- possibly these are population-level averages of these parameters, which can vary between individuals? Lines 187-192 give priors for 'covariate-level parameters', although it is not stated what these covariates could be. From the presentation of the results in the figures, I guess these could be things like: age group, number of prior exposures, VOC, etc. Nevertheless, this presentation is quite shoddy. Then lines 227-234 give the individual-level parameters. Would it be possible to give an example of the full model equation for a particular individual, 'i', in a particular age group, with a given number of exposures, infected with a particular variant, with the individual-level variation included? Or are the covariates fitted one at a time in the models?

We agree that the presentation of the hierarchical structure and the parameters involved could be improved in the supplementary material. We have rewritten the individual-level part of the model description in the revised supplement, including the reviewer's suggestion for a full model equation for a particular individual with particular covariate values. This new write up can be found in the model description section of the revised supplementary material. In the revised write-up of the model description, we now include the model equation for the upwards part of the viral kinetic trajectory for an individual in a specific category for each set of covariates.

Regarding the results presented for things like 'differences in peak Ct value by variant', the authors say very little about the significance (I use the word broadly, rather than in a statistical sense) of their results. Differences (with 95% credible intervals) are presented for the peak Ct value in the (e.g.) Delta group compared to the Omicron BA.1. I suspect the authors consider that if the 95% CrIs don't cross 0 then the difference should be considered 'meaningful'? Is there any reason why the authors didn't do a more formal analysis e.g. with WAIC or LOO-CV? In other words, would a simpler model, without this covariate for variant in the model, explain the data more poorly? The authors may have good reason for not proceeding in this manner, but I'd be interested to hear their justification

We agree with the reviewer’s comment that the small effect size estimates suggest a more formal model selection process would be helpful to justify the inclusion of the covariate category we highlight throughout the study, namely the VOC attributed to each infection episode. In the revised manuscript we have performed an approximate leave-one-out cross validation (LOO-CV) analysis. Specifically, we calculated the estimated (predictive)-log likelihood value (ELPD) using the Pareto-smoothed importance sampling (PSIS) method — implemented by the loo R package — for a number of models, the suggested approach to performing a LOO analysis with complicated models where a full LOO analysis would be computationally expensive. We report their values in an additional table in the supplementary material. We have added the following sentence to the revised manuscript:

*Lastly, Table S7 shows the results from an approximate leave-one-out cross validation performed for seven candidate models, finding similar predictive power between models with different design matrices, justifying the use of the specific set of covariates used in the main analysis.*

And we have added the following section to the revised supplement describing this analysis and the results:

*Leave-one-out model comparison*

*To justify the inclusion of the covariates used in the main analysis (VOC, symptom status, number of exposures and age group), we performed a leave-one-out (LOO) analysis using the loo package in R (ref). The loo package approximates a full LOO analysis (where the model would need to be refit N – 1 times, where N is the total number of Ct values in this case, which would be computationally expensive. The package uses Pareto-smoothed importance sampling, which requires the model to be fit just once for each model type. It gives an expected log-predictive density (ELPD) value for each model fit and a standard error (SE) value associated. Once all model candidates are fit, it gives the ELPD-diff and SE-diff to allow straightforward comparisons between model structures. Table S7 shows the ELPD and associated SEs for all of the model candidates considered.*

*Primarily, we were interested in assessing the inclusion of the VOC covariate, given that there is a strong a priori biological justification for VOC having some effect on shedding, but we want to avoid the risk of overfitting that comes with a more complex model. We found that some models with a single covariate fit at a time perform worse than the model with all covariates and some better, although the differences and standard errors are small between all model candidates. A lower bound for a reliable difference between two model structures is a 4-fold difference between the LOO-IC (which is -2 x ELPD) and the standard error difference, which is not the case between any of our model candidates. Therefore, we conclude that there is no penalty incurred in terms of LOO predictive performance for the more complex VOC model, and potentially even a small advantage. The table reporting the results from the full LOO analysis can be found at Table S7.*

Related to the above point, on line 279 the authors say that individuals with 3 or 4 exposures have 'substantially' higher viral load, compared to individuals with 5 exposures. On what basis is the word 'substantially' used? The 95% CrIs don't seem to support this, if it means that the difference is significant/meaningful. Furthermore, Figure 3C suggests a non-monotonic relationship between the peak Ct value and the number of prior exposures, so this situation seems muddier to me than the authors indicate.

We realise that we could have been clearer in this section. Individuals with 5 exposures had a difference in viral load that is 2.3 (95% CrI: -1—3.7) Ct values larger than those with 4 exposures, indicating a difference between these groups consistent with that which we would expect. However, as the reviewer notes, it is not monotonic between 3, 4, and 5+, which suggests there is likely to be some additional complexity (such as boosting following a recent infection/vaccine in the 3 group relative to the 4) that we cannot untangle here.

We now address this in the updated Discussion:

*Our finding of higher Ct values after 5+ viral exposures (whether vaccine or infection) is consistent with enhanced host immune responses due to previous exposure to spike protein. However, the non-increasing relationship in Ct estimated between 3 and 4 exposures indicates that immunological dynamics, particularly following initial vaccinations and infections at different points in time, may be more complex than can be fully captured in a single exposure total.*

Line 408: I'm not sure I would describe the cohort as 'broadly representative of the UK population', as it seems to solely consist of working age people. It might be reasonable to say 'broadly representative of the working-age UK population'.

We agree that this statement could have been clearer. In the revised manuscript, we have amended this sentence so that it now includes the phrase: *‘broadly representative of the working-age UK population’.*

Supporting Information, line 139: this equation is missing a closing bracket

We thank the reviewer for pointing out this typo. We have corrected it in the revised supplementary material.
