# Supplementary material for: Combined analyses of within-host SARS-CoV-2 viral kinetics and information on past exposures to the virus in a human cohort identifies intrinsic differences of Omicron and Delta variants
Source: PLoS Biol. 2024 Jan 30;22(1):e3002463. doi: 10.1371/journal.pbio.3002463 (PMC10826969; doi:10.1371/journal.pbio.3002463)
Supplement: Attachment — Submitted filename: reply_to_reviewers.docx [file pbio.3002463.s010.docx]

**Reply to Editorial and Reviewers’ comments**

1. ETHICS STATEMENT:

Please include information about the form of consent (written/oral) given for research involving human participants. All research involving human participants must have been approved by the authors' Institutional Review Board (IRB) or an equivalent committee, and all clinical investigation must have been conducted according to the principles expressed in the Declaration of Helsinki.

**Our manuscript includes the following text in the Clinical Cohort Composition subsection of the Methods section. We believe this covers the requirements for an Ethics Statement. If the statement should have its own subsection, we are very happy for this statement to be moved.**

*The Legacy study is a prospective observational cohort study of healthy adult volunteers undergoing weekly occupational health PCR screening for SARS-CoV-2 at University College London Hospitals or at the Francis Crick Institute (NCT04750356) (22,23). The Legacy study was approved by London Camden and Kings Cross Health Research Authority Research and Ethics committee (IRAS number 286469) and is sponsored by University College London Hospitals.*

2. DATA POLICY:

You may be aware of the PLOS Data Policy, which requires that all data be made available without restriction: http://journals.plos.org/plosbiology/s/data-availability. For more information, please also see this editorial: http://dx.doi.org/10.1371/journal.pbio.1001797

Note that we do not require all raw data. Rather, we ask that all individual quantitative observations that underlie the data summarized in the figures and results of your paper be made available in one of the following forms:

A) Supplementary files (e.g., excel). Please ensure that all data files are uploaded as 'Supporting Information' and are invariably referred to (in the manuscript, figure legends, and the Description field when uploading your files) using the following format verbatim: S1 Data, S2 Data, etc. Multiple panels of a single or even several figures can be included as multiple sheets in one excel file that is saved using exactly the following convention: S1_Data.xlsx (using an underscore).

**We have a lot of supplementary data files required to build the 27 supplementary figures. As such, I have added them as a Zenodo repository rather than as Supplementary data files following the guidelines below. We include the both inline in the Supplementary Material and as separate files, as it makes it far easier for the reader to follow if the figures are inline.**

B) Deposition in a publicly available repository. Please also provide the accession code or a reviewer link so that we may view your data before publication.

**We have a Github repository with all of the code publicly available** [**here**](https://github.com/thimotei/legacy_ct_modelling/tree/main) **and a Zenodo repository with all of the large data files to remake all of the figures** [**here**](https://zenodo.org/records/10223386)**.**

Regardless of the method selected, please ensure that you provide the individual numerical values that underlie the summary data displayed in the following figure panels as they are essential for readers to assess your analysis and to reproduce it: Figures 1B, 2, 3, 4, and Supplementary Figures S1, S2, S3, S4, S5, S6, S7, S8, S9, S10, S11, S12, S13, S14, S15, S16, S17, S18, S19, S20, S21, S22, S23, S24, S25AB, S26AB, S27.

**Panel B in Figure 1 does not include any real data. We used Inkscape to draw an illustrative example of two individuals' data, and as such there are no numerical values corresponding to the figure. Due to the illustrative nature of the plot, the Ct value axis doesn’t include numerical values. We believe this is enough, combined with the figure legend explaining that the figure is a schematic of the modelling process, for the reader to understand that there are no accompanying numerical values.**

**The data for all the other figures are saved in the outputs/plot_data .RDS files, each named with the name of the corresponding plot. The only plots which do not have such files is Figure 1. As explained above, Figure 1 is a schematic and therefore doesn’t have any data.**

**These data files are not included on the code repository, as they are too large. However, I have added them to a Zenodo repository** [**here**](https://zenodo.org/records/10223386) **with the DOI: 10.5281/zenodo.10223386. If you clone the Github repository, and download the files from Zenodo, you have everything you need to remake all of the figures**

NOTE: the numerical data provided should include all replicates AND the way in which the plotted mean and errors were derived (it should not present only the mean/average values).

Please also ensure that figure legends in your manuscript include information on where the underlying data can be found, and ensure your supplemental data file/s has a legend.

Please ensure that your Data Statement in the submission system accurately describes where your data can be found.

**We have amended the Data Statement in the manuscript to the following:**

*The dataset containing the Ct values at the three gene targets considered for the 140 infection episodes, symptom onset times and covariate data, along with the code to re-run the analysis can be found at this publicly available repository: https://github.com/thimotei/legacy_ct_modelling. We have achieved the stable release version of this repository on Zenodo with the following DOI: 10.5281/zenodo.10223418. Furthermore, the data required to reproduce all of the figures, table and supplementary figures can be found at this Zenodo repository DOI: 10.5281/zenodo.10223386.*

3. CODE POLICY

Per journal policy, as the code that you have generated is important to support the conclusions of your manuscript, we require that you make it available without restrictions upon publication. Please ensure that the code is sufficiently well documented and reusable, and that your Data Statement in the Editorial Manager submission system accurately describes where your code can be found.

Please note that sole deposition of data or code to GitHub would not be compliant with our policies, as this could be changed after publication (https://journals.plos.org/plosbiology/s/data-availability). However, once the data/code is final, you can archive your publicly available GitHub data to Zenodo. Once you do this, it will also generate a DOI number that you can provide us with. See the process for doing this here: https://docs.github.com/en/repositories/archiving-a-github-repository/referencing-and-citingcontent

**We have a Github repository with all of the code publicly available** [**here**](https://github.com/thimotei/legacy_ct_modelling/tree/main)**, a Zenodo repository of the release of the Github here and a Zenodo repository with all of the large data files to remake all of the figures** [**here**](https://zenodo.org/records/10223386)**.**

4. Please provide a blurb which (if accepted) will be included in our weekly and monthly Electronic Table of Contents, sent out to readers of PLOS Biology, and may be used to promote your article in social media. The blurb should be about 30-40 words long and is subject to editorial changes. It should, without exaggeration, entice people to read your manuscript. It should not be redundant with the title and should not contain acronyms or abbreviations.

*We used a Bayesian model to reveal how a set of key covariates affect viral kinetics at both individual- and population-levels. Covariate categories include: the infecting VOC —including Delta, Omicron-BA.1 and Omicron-BA.2 infected individuals, symptom status, age and number of prior exposures. Furthermore, we jointly infer incubation periods stratified by the same covariate categories and outline how the incubation period is related to the viral kinetics as variants evolved.*

5. We suggest a change in the title: "Combined analyses of within-host SARS-CoV-2 viral kinetics and information on past exposures to the virus in a human cohort identifies intrinsic differences of Omicron and Delta variants".

As you address these items, please take this last chance to review your reference list to ensure that it is complete and correct. If you have cited papers that have been retracted, please include the rationale for doing so in the manuscript text, or remove these references and replace them with relevant current references. Any changes to the reference list should be mentioned in the cover letter that accompanies your revised manuscript.

**We have reviewed the reference list and we believe it is accurate.**

We expect to receive your revised manuscript within two weeks.

**We apologise for the delays. Getting all of the supplementary figures and datasets in order was a time consuming task!**

To submit your revision, please go to https://www.editorialmanager.com/pbiology/ and log in as an Author. Click the link labelled 'Submissions Needing Revision' to find your submission record. Your revised submission must include the following:

- a cover letter that should detail your responses to any editorial requests, if applicable, and whether changes have been made to the reference list

- a Response to Reviewers file that provides a detailed response to the reviewers' comments (if applicable, if not applicable please do not delete your existing 'Response to Reviewers' file.)

- a track-changes file indicating any changes that you have made to the manuscript.

NOTE: If Supporting Information files are included with your article, note that these are not copyedited and will be published as they are submitted. Please ensure that these files are legible and of high quality (at least 300 dpi) in an easily accessible file format. For this reason, please be aware that any references listed in an SI file will not be indexed. For more information, see our Supporting Information guidelines:

<https://journals.plos.org/plosbiology/s/supporting-information>

*Published Peer Review History*

Please note that you may have the opportunity to make the peer review history publicly available. The record will include editor decision letters (with reviews) and your responses to reviewer comments. If eligible, we will contact you to opt in or out. Please see here for more details:

https://blogs.plos.org/plos/2019/05/plos-journals-now-open-for-published-peer-review/

*Press*

Should you, your institution's press office or the journal office choose to press release your paper, please ensure you have opted out of Early Article Posting on the submission form. We ask that you notify us as soon as possible if you or your institution is planning to press release the article.

*Protocols deposition*

To enhance the reproducibility of your results, we recommend that if applicable you deposit your laboratory protocols in protocols.io, where a protocol can be assigned its own identifier (DOI) such that it can be cited independently in the future. Additionally, PLOS ONE offers an option for publishing peer-reviewed Lab Protocol articles, which describe protocols hosted on protocols.io. Read more information on sharing protocols at https://plos.org/protocols?utm_medium=editorial-email&utm_source=authorletters&utm_campaign=protocols

Please do not hesitate to contact me should you have any questions.

Sincerely,

Paula

---

Paula Jauregui, PhD,

Senior Editor,

pjaureguionieva@plos.org,

PLOS Biology

-------------------------------------------------------------------------

Reviewer remarks:

Reviewer #1: All my comments have been addressed. I have no further comment

It would be worth verifying the number of patients in Table 1. Line 155, 135 participants had 1 infection episode, l140 140 participants had 1 infection episode

**We had 135 participants and 140 infection episodes, as five individuals had two infection episodes. We have clarified this at line 162 of the revised manuscript with the new sentence:**

*Five individuals had two recorded reinfections, resulting in a total of 140 infection episodes with at least two positive PCR test results per episode.*

Reviewer #2: Very happy to recommend that this article be accepted. I look forward to seeing it published
